# Reduction in the neuronal surface of post and presynaptic GABA_B_ receptors in the hippocampus in a mouse model of Alzheimer's disease

**DOI:** 10.1111/bpa.12802

**Published:** 2019-12-12

**Authors:** Alejandro Martín‐Belmonte, Carolina Aguado, Rocío Alfaro‐Ruíz, Ana Esther Moreno‐Martínez, Luis de la Ossa, José Martínez‐Hernández, Alain Buisson, Simon Früh, Bernhard Bettler, Ryuichi Shigemoto, Yugo Fukazawa, Rafael Luján

**Affiliations:** ^1^ Synaptic Structure Laboratory Instituto de Investigación en Discapacidades Neurológicas (IDINE) Departamento de Ciencias Médicas Facultad de Medicina Universidad Castilla‐La Mancha Campus Biosanitario, C/ Almansa 14 02008 Albacete Spain; ^2^ Departamento de Sistemas Informáticos Escuela Superior de Ingeniería Informática Universidad de Castilla‐La Mancha 02071 Albacete Spain; ^3^ Grenoble Institut des Neurosciences Université Grenoble Alpes BP 170 Grenoble France; ^4^ Department of Biomedicine Institute of Physiology University of Basel Basel Switzerland; ^5^ Institute of Science and Technology (IST Austria) Am Campus 1 A‐3400 Klosterneuburg Austria; ^6^ Division of Brain Structure and Function Faculty of Medical Science University of Fukui Fukui Japan; ^7^ Life Science Innovation Center University of Fukui Fukui Japan; ^8^ Research Center for Child Mental Development Faculty of Medical Science University of Fukui Fukui Japan; ^9^ Present address: University of the Basque Country (UPV/EHU) 48940 Leioa Spain; ^10^ Present address: Ikerbasque, Basque Foundation for Science 48013 Bilbao Spain

**Keywords:** Alzheimer's disease, electron microscopy, freeze‐fracture, GABA_B_ receptors, hippocampus, immunohistochemistry, ion channels

## Abstract

The hippocampus plays key roles in learning and memory and is a main target of Alzheimer's disease (AD), which causes progressive memory impairments. Despite numerous investigations about the processes required for the normal hippocampal functions, the neurotransmitter receptors involved in the synaptic deficits by which AD disables the hippocampus are not yet characterized. By combining histoblots, western blots, immunohistochemistry and high‐resolution immunoelectron microscopic methods for GABA_B_ receptors, this study provides a quantitative description of the expression and the subcellular localization of GABA_B1_ in the hippocampus in a mouse model of AD at 1, 6 and 12 months of age. Western blots and histoblots showed that the total amount of protein and the laminar expression pattern of GABA_B1_ were similar in APP/PS1 mice and in age‐matched wild‐type mice. In contrast, immunoelectron microscopic techniques showed that the subcellular localization of GABA_B1_ subunit did not change significantly in APP/PS1 mice at 1 month of age, was significantly reduced in the *stratum lacunosum‐moleculare* of CA1 pyramidal cells at 6 months of age and significantly reduced at the membrane surface of CA1 pyramidal cells at 12 months of age. This reduction of plasma membrane GABA_B1_ was paralleled by a significant increase of the subunit at the intracellular sites. We further observed a decrease of membrane‐targeted GABA_B_ receptors in axon terminals contacting CA1 pyramidal cells. Our data demonstrate compartment‐ and age‐dependent reduction of plasma membrane‐targeted GABA_B_ receptors in the CA1 region of the hippocampus, suggesting that this decrease might be enough to alter the GABA_B_‐mediated synaptic transmission taking place in AD.

## Introduction

Alzheimer's disease (AD) is the most prevalent neurodegenerative disease in the elderly population. Alzheimer's disease progression has been associated with a gradual damage in function and structure of the hippocampus, a vulnerable brain region involved in the memory formation and in the cognition. In particular, the CA1 hippocampal region is one of the most affected brain areas in AD, suffering a variety of neuronal alterations, including dendritic changes in pyramidal cells and pronounced loss of neurons 55, 56. The three major neuropathology hallmarks of AD are extracellular amyloid plaques containing amyloid β (Aβ) peptides derived from amyloid precursor protein (APP), neurofibrillary tangles of aggregated hyperphosphorylated tau in neurons and synapse loss 5. The progressive increase in synapse loss correlates with cognitive decline and is proposed to underlie learning and memory deficits in AD 21.

Amyloid β (Aβ) has been implicated in the pathogenesis of AD by creating a microenvironment that damages the dendritic spines, which represent the major postsynaptic elements of excitatory synapses in the cerebral cortex 16, and are fundamental to memory formation, learning and cognition 30. Growing evidence supports that structural plasticity impairments on excitatory synapses are linked to a gross disruption of the glutamatergic system 36, 41, 55, 58, 59. Thus, N‐methyl‐D‐aspartate (NMDA) receptors activation and removal of AMPA receptors have been implicated in AD‐related synaptic dysfunctions 20, 59. The neurotransmitter γ‐aminobutyric acid (GABA) acts through GABA_A_ and GABA_B_ receptors to inhibit neurons 3, 13. While alterations in the expression of GABA_A_ receptor subunits in the AD hippocampus differ 28 alterations in GABA_B_ receptor expression are less well understood and only recently it has been shown a direct molecular and functional link between APP and GABA_B_ receptors 9, 45, 47.

GABA_B_ receptors have modulatory actions on neuronal excitability and neurotransmitter release, and are involved in a number of physiological and pathophysiological processes 3, including AD. Two subunits, GABA_B1_ and GABA_B2_, are required to form functional receptors 26, 37. Autoradiographic, *in situ* hybridisation and immunohistochemical studies showed that the hippocampus expresses a high‐density of GABA_B_ receptors 4, 6, 7, 12, 25, 32. Immunoelectron microscopic studies demonstrated an association of GABA_B_ receptors with postsynaptic and presynaptic sites at excitatory synapses in the hippocampus, with a particularly high abundance in the dendritic spines of pyramidal cells 8, 27, 32, 53. Since dendritic spines and excitatory synapses are lost in AD it is expected that both the distribution and functions of GABA_B_ receptors are affected. However, it remains unclear how these receptors reorganize at the surface of hippocampal neurons in AD brains. For this purpose, our work adopted transgenic mice overexpressing mutant familial AD genes [amyloid‐β protein precursor (AβPP), presenilin‐1 (PS1)‐dE9], considered one of the most relevant animal models of AD. These animals show Aβ deposition by 4 months with a progressive increase in senile plaque number up to 12 months and cognitive impairments 14, 15.

To identify possible alterations in the expression and subcellular localization of GABA_B_ receptors in the APP/PS1 mice we used western blots, histoblots and high‐resolution immunohistochemical techniques in combination with quantitative approaches at different ages. Here we provide evidence for a significant reduction in the plasma membrane expression of GABA_B_ receptors in CA1 pyramidal cells in the APP/PS1 AD mouse model.

## Material and Methods

### Animals

The mouse line used for this study (APP/PS1; hemizygote animals) expressed Mo/Hu APP695swe construct in conjunction with the exon 9‐deleted variant of human presenilin 1 (PS1‐dE9) 46. Control mice were age‐matched littermates without the transgene (wild type). Mice of all genotypes were aged to 1, 6 and 12 months before use in a battery of biochemical and morphological experiments. For each age and genotype, four mice were used for western blot, four mice were used for histoblot techniques, four mice were used for SDS‐FRL techniques and three mice were used for pre‐embedding immunoelectron microscopic analyses. All mice were obtained from the Animal House Facility of the University of Castilla‐La Mancha (Albacete, Spain). Animals were housed in cages of 2 or more mice, maintained on a 12 hour light/12 hour dark cycle at 24°C and received food and water *ad libitum*. Care and handling of animals prior to and during experimental procedures were in accordance with Spanish (RD 1201/2015) and European Union regulations (86/609/EC), and the protocols were approved by the local Animal Care and Use Committee.

For western blots, the animals were deeply anesthetized by intraperitoneal injection of ketamine/xylazine 1:1 (0.1 mL/kg b.w.), the hippocampus was extracted, frozen rapidly in liquid nitrogen and stored at −80°C. For histoblotting, the animals were deeply anesthetized by intraperitoneal injection of ketamine/xylazine 1:1 (0.1 mL/kg b.w.), the brains were quickly frozen in liquid nitrogen and stored at −80°C. For immunohistochemistry at the light and electron microscopic levels, animals were deeply anesthetized by intraperitoneal injection of ketamine‐xylazine 1:1 (0.1 mL/kg) and transcardially perfused with ice‐cold fixative containing: (i) 2% paraformaldehyde in 0.1 M phosphate buffer (PB, pH 7.4) for 12 min (for SDS‐FRL technique), or (ii) 4% paraformaldehyde, 0.05% glutaraldehyde and 15% (v/v) saturated picric acid made up in 0.1 M phosphate buffer (PB), pH 7.4 (for light microscopy and pre‐embedding immunogold techniques). After perfusion, brains were removed and immersed in the same fixative for 2 h or overnight at 4°C. Tissue blocks were washed thoroughly in 0.1 M PB. Coronal sections (60 µm thickness) were cut on a Vibratome (Leica V1000).

### Antibodies and chemicals

An affinity‐purified polyclonal antibody against GABA_B1_ (B17, aa. 901‐960 of rat GABA_B1_) was raised in rabbit, and used for immunohistochemical techniques at the light and electron microscopic levels. The characteristics and specificity of the anti‐GABA_B1_ antibody have been described elsewhere 26, 35, 53. For western blots and histoblots, a mouse monoclonal anti‐GABA_B1_ (sc‐166408; D‐2, aa 929‐958 of rat C‐terminus of GABA_B1_) antibody was used (Santa Cruz, CA, USA), and we provided additional information on its specificity. A monoclonal anti‐α‐Tubulin (DM1A; ref CP06) was obtained from Millipore (Millipore Corporation, Burlington, MA, USA). An affinity‐purified polyclonal antibody against β‐amyloid (ref #2454, detecting human Aβ‐40 and Aβ‐42 peptides) raised in rabbit was obtained from Cell Signalling Technology (Leiden, The Netherlands).

The secondary antibodies used were as follows: goat anti‐mouse IgG‐horseradish peroxidase (1:2000; Santa Cruz Biotechnology, Santa Cruz, CA, USA), goat anti‐rabbit IgG‐horseradish peroxidase (1:15 000; Pierce, Rockford, USA), alkaline phosphatase AP‐goat anti‐mouse IgG (H + L) and AP‐goat anti‐rabbit IgG (H + L) (1:5000; Invitrogen, Paisley, UK), goat anti‐rabbit IgG coupled to 1.4 nm gold (1:100; Nanoprobes Inc., Stony Brook, NY, USA) and anti‐rabbit IgG conjugated to 10 nm gold particles (1:100; British Biocell International, Cardiff, UK).

### Western blots

Hippocampi were homogenized in 50 mM Tris Base, pH 7.4 and Protease Inhibitor Cocktail (Thermo Scientific, Pierce, Rockford, USA) with a pestle motor (Sigma‐Aldrich). The homogenized tissue was centrifuged 10 min at 1000 × *g* at 4°C and the supernatant was centrifuged 30 min at 12000 × *g* (Centrifuge 5415R, Eppendorf, Hamburg, Germany) at 4°C, the pellet containing the membrane extracts were resuspended in 50 mM Tris Base, pH 7.4 and Protease Inhibitor Cocktail (Thermo Scientific, Pierce, Rockford, USA). The protein content of each membrane extract was determined by BCA Protein Assay Kit (Thermo Scientific). Twenty‐five micrograms of membrane protein were loaded in Sodium dodecyl sulfate polyacrylamide gel electrophoresis (SDS/PAGE) using 7.5% polyacrylamide with loading sample buffer (0,05 M Tris pH 6.8, 2% SDS, 10% glycerol, 0,05% *β*‐mercaptoethanol and 0,001% bromophenol blue). The proteins were transferred to PVDF membranes using a semidry transfer system and immunoblotted with anti‐GABA_B1_ (1:500) and anti‐α‐Tubulin (1:1000). Protein bands were visualized after application of a mouse IgG kappa‐binding protein coupled to horseradish peroxidase (1:1000) using the enhanced chemiluminescence (ECL) blotting detection kit (SuperSignal West Dura Extended Duration Substrate, Pierce, Rockford, USA). Blots were captured and quantified by densitometry using a LAS4000 MINI (Fujifilm, Japan). A series of primary and secondary antibody dilutions and incubation times were used to optimize the experimental conditions for the linear sensitivity range, confirming that our labeling was well below the saturation levels.

### Histoblotting

The regional distribution of GABA_B1_ was analysed in rodent brains, using an *in situ* blotting technique (histoblot) 1, 52. Briefly, horizontal cryostat sections (10 µm) from mouse brain were apposed to nitrocellulose membranes moistened with 48 mM Tris‐base, 39 mM glycine, 2% (w/v) sodium dodecyl sulphate and 20% (v/v) methanol for 15 min at room temperature (~20°C). After blocking in 5% (w/v) nonfat dry milk in phosphate‐buffered saline, nitrocellulose membranes were treated with DNAse I (5 U/mL), washed and incubated in 2% (w⁄v) sodium dodecyl sulphate and 100 mm β‐mercaptoethanol in 100 mM Tris–HCl (pH 7.0) for 60 min at 45°C to remove adhering tissue residues. After extensive washing, the blots were reacted with affinity‐purified anti‐GABA_B1_ antibodies (0.5 mg⁄mL) in blocking solution overnight at 4°C. The bound primary antibodies were detected with alkaline phosphatase‐conjugated anti‐rabbit IgG secondary antibodies 52. A series of primary and secondary antibody dilutions and incubation times were used to optimize the experimental conditions for the linear sensitivity range of the alkaline phosphatase reactions. To compare the expression levels of GABA_B1_ between the two genotypes (wild type and APP/PS1) and ages (1, 6 and 12‐months), all nitrocellulose membranes were processed in parallel, and the same incubation time for each reagent was used for the antibody.

To facilitate the identification of brain regions, structures and cell layers, adjacent cryostat sections were stained with cresyl violet for the two genotypes (wild type and APP/PS1) and ages (1, 6 and 12‐months; not shown). Digital images were acquired by scanning the nitrocellulose membranes using a desktop scanner (HP Scanjet 8300). Image analysis and processing were performed using the Adobe Photoshop software (Adobe Systems, San Jose, CA, USA) as described previously 10. All of the images were processed with the same equipment in the same way to allow comparison of the intensity of grayscale images between experimental groups and in different brain regions. The pixel density (arbitrary units) of immunoreactivity was measured using open circular cursors with a diameter of 0.10 mm. The cursors were placed in different brain regions identified based on the adjacent cresyl violet‐stained sections 10. We used background correction to eliminate the potential differences in optical densities across different sections in different experiments. The average of eight background determinations carried out near the brain protein‐containing areas of the immunostained nitrocellulose membranes was subtracted from the average pixel densities measured within the brain regions. Following background corrections, the average pixel density for the whole region from one animal counted as one ‘n’. Under these conditions, labeling performed on different days produced very consistent results. Data were analyzed and plotted using the software Analysis (GraphPad Prism, San Diego, USA).

### Immunohistochemistry for light microscopy

Immunohistochemical reactions at the light microscopic level were carried out using the immunoperoxidase method as described earlier 34. Briefly, sections were incubated in 10% normal goat serum (NGS) diluted in 50 mM Tris buffer (pH 7.4) containing 0.9% NaCl (TBS), with 0.2% Triton X‐100, for 1 h. Sections were incubated in anti‐GABA_B1_ (1–2 µg/mL diluted in TBS containing 1% NGS) or in anti‐β amyloid (1–2 µg/mL diluted in TBS containing 1% NGS), followed by incubation in biotinylated goat anti‐rabbit IgG (Vector Laboratories, Burlingame, CA) diluted 1:200 in TBS containing 1% NGS. Sections were then transferred into avidin–biotin–peroxidase complex (ABC kit, Vector Laboratories). Bound peroxidase enzyme activity was revealed using 3,3′‐diaminobenzidine tetrahydrochloride (DAB; 0.05% in TB, pH 7.4) as the chromogen and 0.01% H_2_O_2_ as the substrate. Finally, sections were air‐dried and mounted prior to observation in a Leica photomicroscope (DM2000) equipped with differential interference contrast optics and a digital imaging camera.

### Immunohistochemistry for electron microscopy

Immunohistochemical reactions at the electron microscopic level were carried out using the pre‐embedding immunogold and the SDS‐digested freeze‐fracture replica labeling (SDS‐FRL) methods as described earlier 33, 34, 50.

*Pre‐embedding immunogold method*. Briefly, free‐floating sections obtained from the two genotypes (WT and APP/PS1) and three ages (1, 6 and 12‐months) were incubated in parallel in 10% (v/v) NGS diluted in TBS. Sections were then incubated in anti‐GABA_B1_ antibodies (3‐5 μg⁄mL diluted in TBS containing 1% (v/v) NGS), followed by incubation in goat anti‐rabbit IgG coupled to 1.4 nm gold (Nanoprobes Inc., Stony Brook, NY, USA), respectively. Sections were postfixed in 1% (v/v) glutaraldehyde and washed in double‐distilled water, followed by silver enhancement of the gold particles with an HQ Silver kit (Nanoprobes Inc.). Sections were then treated with osmium tetraoxide (1% in 0.1 M phosphate buffer), block‐stained with uranyl acetate, dehydrated in graded series of ethanol and flat‐embedded on glass slides in Durcupan (Fluka) resin. Regions of interest were cut at 70–90 nm on an ultramicrotome (Reichert Ultracut E, Leica, Austria) and collected on single slot pioloform‐coated copper grids. Staining was performed on drops of 1% aqueous uranyl acetate followed by Reynolds's lead citrate. Ultrastructural analyses were performed in a JEOL‐1010 electron microscope.

*SDS‐FRL technique*. Animals were anesthetized with sodium pentobarbital (50 mg/kg, i.p.) and perfused transcardially with 25 mM PBS for 1 min, followed by perfusion with 2% paraformaldehyde in 0.1 M phosphate buffer (PB) for 12 min. The hippocampi were dissected and cut into sagittal slices (130 µm) using a Microslicer (Dosaka, Kyoto, Japan) in 0.1 M PB. Next, we trimmed hippocampal slices containing the CA region and immersed them in graded glycerol of 10%–30% in 0.1 M PB at 4°C overnight. Slices were frozen using a high‐pressure freezing machine (HPM010, BAL‐TEC, Balzers). Slices were then fractured into two parts at ‐120°C and replicated by carbon deposition (5 nm thick), platinum (60° unidirectional from horizontal level, 2 nm) and carbon (15‐20 nm) in a freeze‐fracture replica machine (BAF060, BAL‐TEC, Balzers). Replicas were transferred to 2.5% SDS and 20% sucrose in 15 mM Tris buffer (pH 8.3) for 18 h at 80°C with shaking to dissolve tissue debris. The replicas were washed three times in 50 mM Tris‐buffered saline (TBS, pH 7.4), containing 0.05% bovine serum albumin (BSA) and then blocked with 5% BSA in the washing buffer for 1 h at room temperature. Next, the replicas were washed and reacted with a polyclonal rabbit antibody for GABA_B1_ (5μg/mL) at 15°C overnight. Following three washes in 0.05% BSA in TBS and blocking in 5% BSA/TBS, replicas were incubated in secondary antibodies conjugated with 10 nm gold particles overnight at room temperature. When the primary antibody was omitted, no immunoreactivity was observed. After immunogold labeling, the replicas were immediately rinsed three times with 0.05% BSA in TBS, washed twice with distilled water and picked up onto grids coated with pioloform (Agar Scientific, Stansted, Essex, UK).

### Quantification and analysis of SDS‐FRL data

The labeled replicas were examined using a transmission electron microscope (JEOL‐1010) and photographed at magnifications of 60,000, 80,000 and 100,000. All antibodies used in this study were visualized by immunoparticles on the protoplasmic face (P‐face), consistent with the intracellular location of their epitopes. Nonspecific background labeling was measured on E‐face surfaces in wild‐type mice. Digitised images were then modified for brightness and contrast using Adobe PhotoShop CS5 (Mountain View, CA, USA) to optimize them for quantitative analysis. The quantitative analyses were done using the software GPDQ (*Gold Particle Detection and Quantification*) developed recently to perform automated and semiautomated detection of gold particles present in a given compartment of neurons 33.

#### Density gradient of GABA_B1_ along the membrane surface

The procedure was similar to that used previously 33. Briefly, immunogold labeling for GABA_B1_ was achieved from replicas containing all layers of the CA1 region, so that the laminar distribution could be compared under identical conditions for each animal and experimental group. Quantitative analysis of immunogold labeling for GABA_B1_ was performed on ten different dendritic compartments of pyramidal cells in all dendritic layers of the CA1 region and in somata of pyramidal cells in the *stratum pyramidale*. The dendritic compartments analysed were the main dendritic shaft (apical dendrites), spiny branchlets (oblique dendrites) and dendritic spines. Oblique dendrites were identified based on their small diameter and the presence of at least one emerging spine from the dendritic shaft. Dendritic spines were considered as such if: (i) they emerged from a dendritic shaft or (ii) they opposed an axon terminal. Axon terminals were identified based on: (i) the concave shape of the P‐face and the accumulation of intramembrane particles (IMPs) on the opposing exoplasmic‐face (E‐face) of a spine or dendrite; or (ii) the presence of synaptic vesicles on their cross‐fractured portions. Non‐specific background labeling was measured on E‐face structures surrounding the measured P‐faces. Images of the identified compartments were selected randomly over the entire dendritic tree of CA1 pyramidal cells and then captured with an ORIUS SC1000 CCD camera (Gatan, Munich, Germany). The area of the selected profiles and the number of immunoparticles were measured using our GPDQ software 33. Immunoparticle densities were presented as mean ± SEM between animals. Statistical comparisons were performed with GraphPad Prism 5 software (La Jolla, CA, USA).

#### Number of GABA_B1_ immunoparticles in clusters along the membrane surface

To determine the number of GABA_B1_ immunoparticles composing the clusters that are present in different compartments of CA1 pyramidal cells in the two genotypes (wild type and APP/PS1) and the three ages (1, 6 and 12‐months), we used the same set of images for the above analysis and the GPDQ software. The software reports the number of particles, their area (as the area inside the convex hull of the particles in the cluster) or the distance to the nearest cluster of particles. By default, the software uses the minimum number of particles in a cluster fixed to three, so all clusters with one or two particles are discarded. More information in all parameters that need to be set in the software is detailed elsewhere 33. The data were expressed as the percentage of clusters with distinct number of immunoparticles per cluster, in different compartments both in wild‐type and APP/PS1 mice.

### Quantification and analysis of pre‐embedding immunogold data

To establish the relative abundance of GABA_B1_ immunoreactivity in different compartments of CA1 pyramidal cells between the two genotypes (wild type and APP/PS1) and the three ages (1, 6 and 12‐months), we used 60‐μm‐thick coronal slices processed for pre‐embedding immunogold immunohistochemistry. The procedure was similar to that used previously 34. Briefly, for each of three adult animals, three samples of tissue were obtained for the preparation of embedding blocks, thus using in total nine blocks. To minimize false negatives, electron microscopic serial ultrathin sections were cut close to the surface of each block, as immunoreactivity decreased with depth. We estimated the quality of immunolabeling by always selecting the areas with optimal gold labeling at approximately the same distance from the cutting surface. Randomly selected areas were then photographed from the selected ultrathin sections and used with final magnification between 30 000 and 50 000×. Quantification of immunogold labeling was carried out in reference areas of the CA1 region totaling approximately 2500 µm^2^. We counted immunoparticles identified in each reference area and present in different subcellular compartments: dendritic spines, dendritic shafts and axon terminals. The data were expressed as a percentage of immunoparticles in each subcellular compartment, both in the plasma membrane and at intracellular sites.

### Controls

To test method specificity in the procedures for electron microscopy, the primary antibody was either omitted or replaced with 5% (v/v) normal serum of the species of the primary antibody, resulting in total loss of the signal. For the pre‐embedding technique, labelling patterns were also compared with those obtained with Calbindin (polyclonal rabbit anti‐Calbindin D‐9k CB9; Swant, Marly, Switzerland); only the antibodies against GABA_B1_ consistently labeled the plasma membrane. To test method specificity in the procedures for western blots and histoblots, antisera against GABA_B1_ was tested on hippocampal membranes and brain sections, respectively, of GABA_B1_ knockout mice. In our samples, the immunolabeling signal was completely absent in the knockout mice, while a strong signal was present in wild‐type mice.

### Data analysis

Statistical analyses for morphological data were performed using SigmaStat Pro (Jandel Scientific) and data were presented as mean ± SEM unless indicated otherwise. Statistical significance was defined as *P* < 0.05. The statistical evaluation of the immunogold densities was performed using the two‐way ANOVA test and further compared with the Bonferroni *post hoc* test.

## Results

### Similar brain expression of GABA_B_ receptors in control and APP/PS1 mice

**Figure 1:**
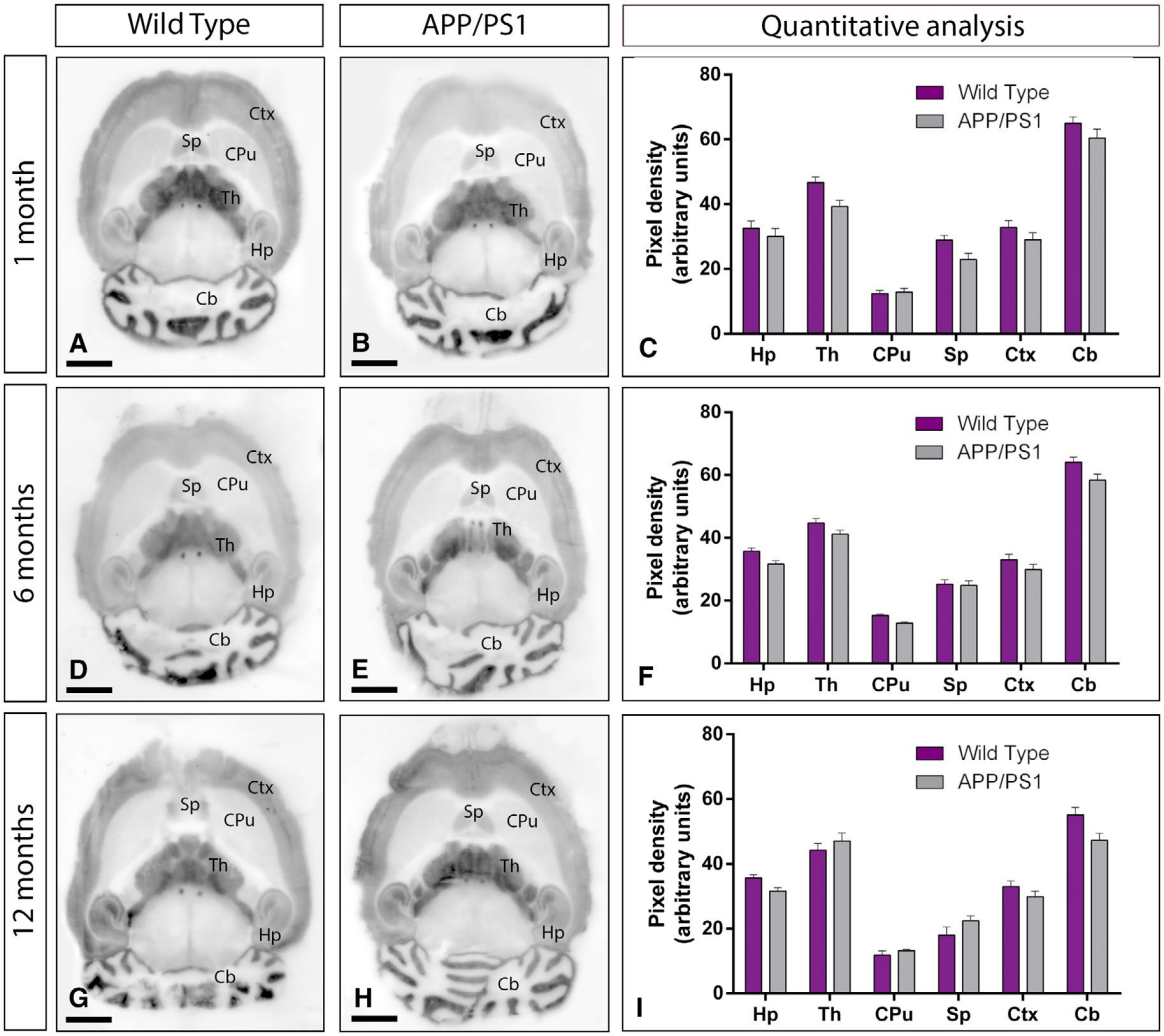
*Regional expression of GABA_B1_ in the brain in wild‐type and APP/PS1 mice*. **A‐I**. The expression of the GABA_B1_ protein was visualized in histoblots of horizontal brain sections at 1, 6 and 12 months of age in wild‐type and APP/PS1 mice using an affinity‐purified anti‐GABA_B1_ antibody. GABA_B1_ expression in different brain regions was determined by densitometric analysis of the scanned histoblots (panels **C**, **F** and **I**). The strongest GABA_B1_ expression was detected in the cerebellum (Cb) and thalamus (Th), with moderate expression in the hippocampus (Hp), cortex (Ctx) and septum (Sp). The weakest expression level was detected in the caudate putamen (CPu). Densitometric analysis showed no differences in GABA_B1_ expression in APP/PS1 mice compared to age‐matched wild type controls. Error bars indicate SEM. Scale bars: 0.2 cm.

We first determined whether the GABA_B_ receptor expression was impaired in the brain of APP/PS1 mice at time points when Aβ deposition is observed. We selected ages with (i) no sign of pathology (1 month; Figure 1A‐C), (ii) starting Aβ deposition (6 months; Figure 1D‐F) and (iii) cognitive deficits, severe synapse loss and widespread Aβ deposition (12 months; Figure 1G‐I). To analyse region‐dependent alterations in GABA_B_ receptor expression in the brain of APP/PS1 and age‐matched wild‐type mice, we used a GABA_B1_ subunit‐specific antibody in conventional histoblots 33.

In the brain, the overall expression of GABA_B1_ revealed marked region‐specific differences at all ages analysed, with very similar expression patterns in wild type and APP/PS1 mice (Figure 1A‐I). In wild‐type mice of the three ages analyzed, immunoreactivity for GABA_B1_ was widely distributed in the brain, with strongest immunoreactivities in the cerebellum and thalamus, moderate labeling in the hippocampus, cortex and septum and weak labeling in the caudate putamen and midbrain nuclei, including the inferior and superior colliculi (Figure 1A,D,G). This expression pattern was very similar in the brain of APP/PS1 mice at 1 (Figure 1B), 6 (Figure 1E) and 12 months (Figure 1H) of age. Quantitative analyses was performed to compare the protein densities for 1, 6 and 12 months of age revealed that GABA_B1_ expression levels were similar between wild‐type and APP/PS1 mice (Figure 1C,F,I).

### Similar hippocampal expression of GABA_B_ receptors in control and APP/PS1 mice

**Figure 2:**
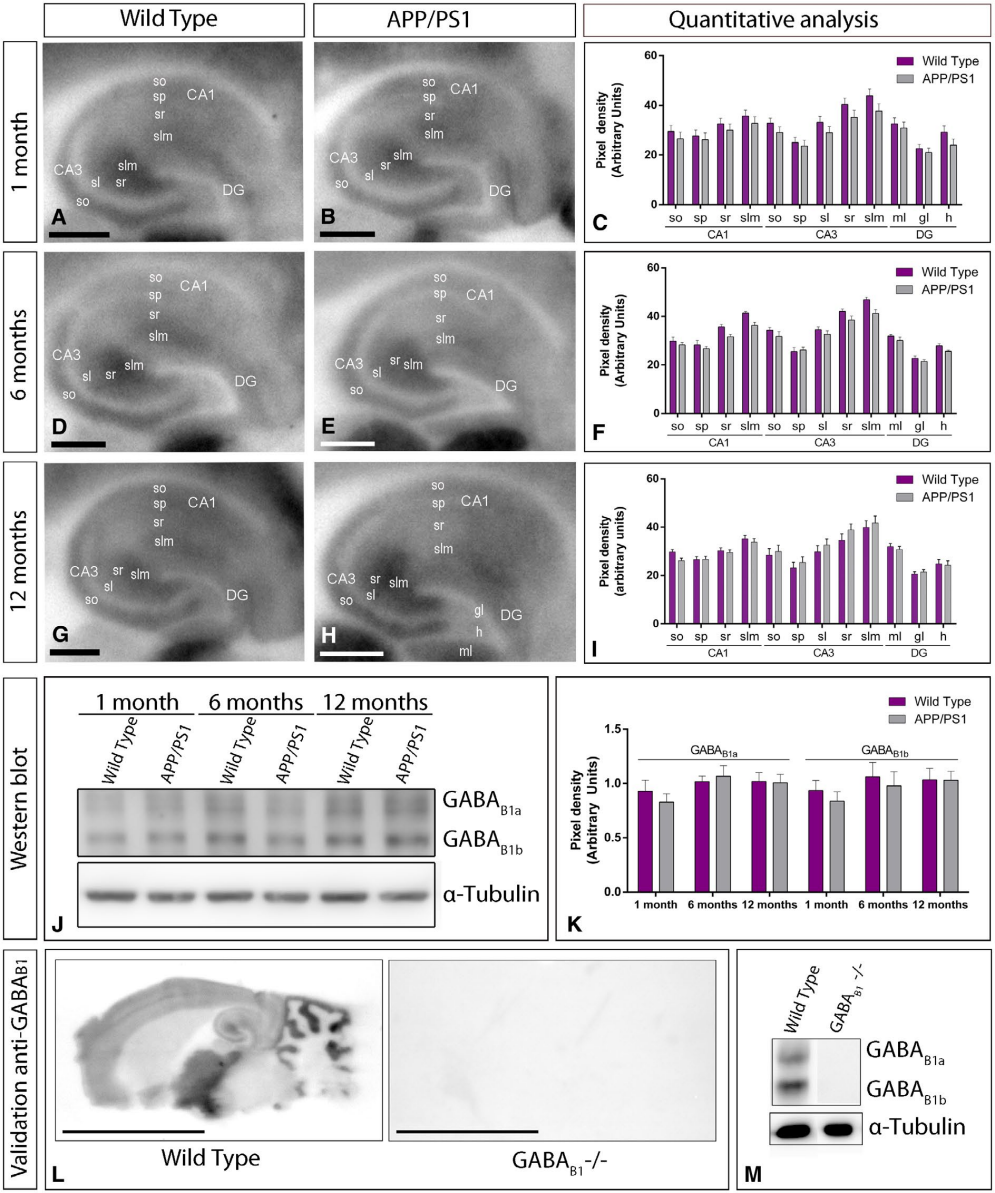
*Hippocampal expression and distribution of GABA_B1_ in wild‐type and APP/PS1 mice*. **A‐I**. The expression of the GABA_B1_ protein was visualized in histoblots of horizontal brain sections at 1, 6 and 12 months of age in wild‐type and APP/PS1 mice using an affinity‐purified anti‐GABA_B1_ antibody. GABA_B1_ expression in different hippocampal subfields and dendritic layers was determined by densitometric analysis of the scanned histoblots. GABA_B1_ expression was moderate to strong in all dendritic layers of the CA1 and CA3 region and dentate gyrus, with the *stratum lacunosum‐moleculare* (slm) of CA3 showing the highest expression level. Densitometric analysis showed no differences in GABA_B1_ expression in APP/PS1 mice compared to age‐matched wild type controls. **J,K**. Western blots showing the expression of the GABA_B1_ protein in the hippocampus at 1, 6 and 12 months of age in wild‐type and APP/PS1 mice. Crude membrane preparations were subjected to 7.5% SDS‐PAGE, transferred on to polyvinylidene difluoride membranes. They were reacted with an anti‐pan GABA_B1_ antibody, which recognised both GABA_B1a_ and GABA_B1b_ subunits with estimated molecular masses of 130 and 100 kDa, respectively. The developed immunoblots were scanned and densitometric measurements from five independent experiments were averaged together to compare the protein densities for each age and experimental group. Quantification of GABA_B1a_ and GABA_B1b_ normalised to α‐tubulin and expressed as pixel density showed no differences in APP/PS1 mice compared to age‐matched wild type controls. Error bars indicate SEM. **L,M**. Characterization of the monoclonal anti‐GABA_B1_ in the mouse brain. The pattern of GABA_B1_ expression observed in the wild type using histoblot disappeared completely in the brain of GABA_B1_ null mice (GABA_B1_‐/‐). Using western blots, immunolabeling signal was abolished in the GABA_B1_‐/‐ mice, while strong bands for GABA_B1a_ and GABA_B1b_ subunits were present in the wild type. Scale bars: **A,B,D,E,G,H**, 0.05 cm; **L**, 0.5 cm. [Correction added on 6 March 2020, after first online publication: The western blot image in Figure 2 has been amended to a version without splicing.]

We next focused on the hippocampus, considered as one of the most vulnerable brain regions affected in AD 56, 57 and explored the laminar expression pattern of GABA_B_ receptors. Using the histoblot technique, we observed that GABA_B1_ was widely expressed in all hippocampal subfields and dendritic layers at the three ages of wild‐type and APP/PS1 mice (Figure 2A‐I). In the CA1 region of wild‐type mice, at 1, 6 and 12 months of age, GABA_B1_ expression was moderate to strong, with the *strata oriens* and *radiatum* showing the lowest and the *stratum lacunosum‐moleculare* showing the highest expression levels (Figure 2A,D,G). The CA3 region of wild‐type mice exhibited higher GABA_B1_ expression level than the CA1 region, with *the stratum lacunosum‐moleculare* showing the highest expression level within the hippocampus, followed by the *stratum radiatum* (Figure 2A,D,G). The *stratum lucidum* showed weak expression level throughout (Figure 2A,D,G). In the dentate gyrus of wild type, GABA_B1_ immunostaining was weak in the hilus and moderate in the molecular layer (Figure 2A,D,G). The *stratum pyramidale* of the CA1 and CA3 regions and the granule cell layer of the dentate gyrus showed the weakest GABA_B1_ expression (Figure 2A,D,G). The expression pattern of wild‐type mice was similar to that of APP/PS1 mice at 1 (Figure 2B), 6 (Figure 2E) and 12 months (Figure 2H) of age. Quantitative analyses of protein densities performed at the three ages confirmed that the expression levels of GABA_B1_ in all subfields and dendritic layers analysed was unchanged between wild‐type and APP/PS1 mice (Figure 2C,F,I).

To corroborate the above histoblot data, we further evaluated the expression of GABA_B1_ in the hippocampus using western blot. The mouse pan anti‐GABA_B1_ subunit antibody revealed two immunoreactive products with estimated molecular masses of 130 and 100 kDa (Figure 2J), corresponding to the GABA_B1a_ and to the GABA_B1b_ proteins, respectively 25. Since GABA_B1a_ and GABA_B1b_ subunits show a differential compartmentalization in hippocampal neurons 53, both subunits were quantified. The levels of GABA_B1a_ and GABA_B1b_ subunit proteins were unchanged in APP/PS1 compared to age‐matched wild‐type mice (Figure 2J,K). As a control, to validate the specificity of the immunoreactions, GABA_B1_ knockout (KO) mice 18 were used. The pattern of immunolabeling for GABA_B1_ observed in the brain of wild‐type mice using western blots and histoblots was completely missing in that of the corresponding GABA_B1_ knockout mice (GABA_B1_ ‐/‐ brains) (Figure 2L,M), thus demonstrating the specificity of the antibody.

### Similar cellular distribution of GABA_B_ receptors in control and APP/PS1 mice

**Figure 3:**
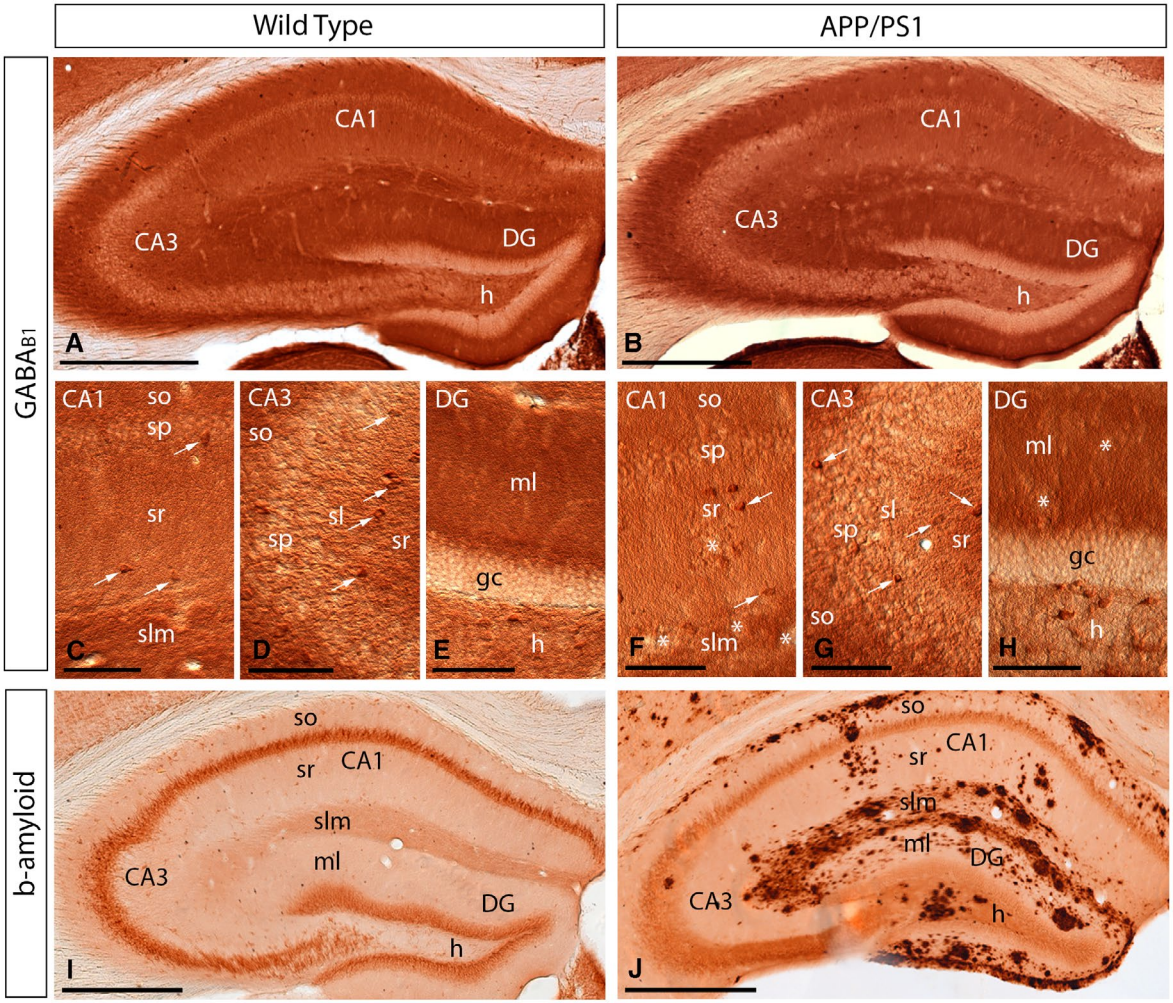
*Regional and cellular distribution of GABA_B1_ in wild‐type and APP/PS1 mice*. **A‐H**. Immunoreactivity for GABA_B1_ in the hippocampus of wild‐type and APP/PS1 mice at 12 months of age using a pre‐embedding immunoperoxidase method at the light microscopic level. In the CA1 and CA3 regions and dentate gyrus (DG), GABA_B1_ immunoreactivity was very similar both in the wild‐type and the APP/PS1 mice, regardless of accumulation of amyloid plaques (asterisks). Labeling for GABA_B1_ showed the highest intensity in the *stratum lacunosum‐moleculare* (slm) and molecular layer (ml) and weaker in the *strata oriens* (so) and *radiatum* (sr). Immunoreactivity for GABA_B1_ was also detected in interneurons throughout all layers (white arrows), with similar distribution pattern and labeling intensity in wild‐type and APP/PS1 mice. **I,J**. Immunoreactivity for β‐amyloid in wild‐type and APP/PS1 mice at 12 months of age, showing high accumulation of Aβ throughout all layers of the hippocampus. *Abbreviations*: CA1 region of the hippocampus; CA3, CA3 region of the hippocampus; DG, dentate gyrus; so, *stratum oriens*; sp, *stratum pyramidale*; sr, *stratum radiatum*; slm, *stratum lacunosum‐moleculare*; ml, molecular layer; gc, granule cell layer; h, hilus. Scale bars: **A,B,I,J** 200 µm; **C‐H**, 100 µm.

Given the similar laminar expression of GABA_B1_ in the hippocampus at the three ages analysed, we next sought to investigate the cellular distribution of the receptor at 12 months of age, using light microscopy immunohistochemical techniques. Both in wild‐type and APP/PS1 mice, immunoreactivity for GABA_B1_ in the CA1 and CA3 regions was the highest in the *stratum lacunosum‐moleculare* and weaker in the *strata oriens* and *radiatum* (Figure 3A‐H). In the dentate gyrus, immunoreactivity for GABA_B1_ was strongest in the molecular layer and weakest in the hilus (Figure 3A‐H). In the *stratum pyramidale* of the CA1 and CA3 regions and the granule cell layer of the dentate gyrus immunoreactivity for GABA_B1_ was weakest in the hippocampus, but again very similar between wild‐type and APP/PS1 mice (Figure 3C‐H). At the cellular level, immunoreactivity for GABA_B1_ was observed in somata of CA1 and CA3 pyramidal cells and granule cells in the dentate gyrus, as well as in somata of some interneurons scattered throughout the hippocampus (Figure 3C‐H). The accumulation of Aβ was very high throughout the hippocampus and particularly high in the *stratum lacunosum‐moleculare* of the CA1 region and all layers of the dentate gyrus compared to age‐matched wild‐type mice (Figure 3I,J). Regardless of this massive accumulation of Aβ, the cellular distributions for GABA_B1_ were very similar in wild type compared to APP/PS1 mice.

### Altered distribution of GABA_B1_ at the plasma membrane of 6 months old APP/PS1 mice

**Figure 4:**
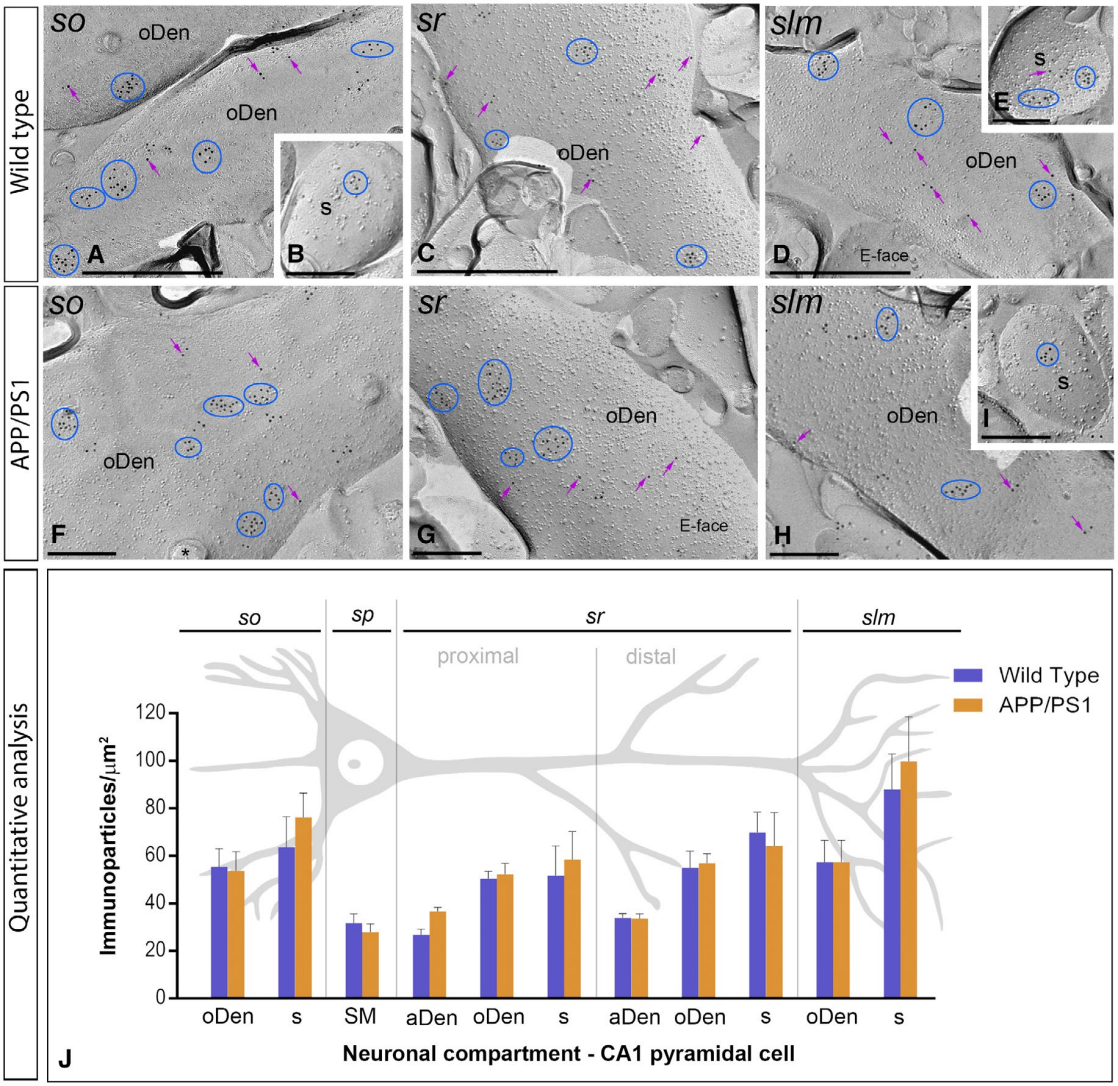
*Subcellular localization of GABA_B1_ in the hippocampus in wild‐type and APP/PS1 mice at 1 month*. Electron micrographs obtained from different strata of the CA1 region showing immunoparticles for GABA_B1_ along the membrane surface of pyramidal cells, as detected using the SDS‐FRL technique. **A‐H**. In wild type and APP/PS1, clusters of GABA_B1_ immunoparticles (blue ellipses/circles) associated with the P‐face were detected in dendritic shafts, illustrated here for oblique dendrites (oDen) and dendritic spines (s) of pyramidal cells in all strata of the CA1 region. Lower density of immunoparticles for GABA_B1_ was also detected scattered (purple arrows) outside the clusters. Fractured spine necks are indicated with asterisks (*). The E‐face is free of any immunolabeling. **J**. Quantitative analysis of GABA_B1_ immunogold labeling in eleven neuronal compartments. The density gradient of GABA_B1_ immunoparticles along the membrane surface of CA1 pyramidal cells was very similar between wild‐type and APP/PS1 mice. Error bars indicate SEM. Abbreviations: *so*, *stratum oriens*; *sp*, *stratum pyramidale*; *sr*, *stratum radiatum*; *slm*, *stratum lacunosum‐moleculare*; aDen, apical dendrite; oDen, oblique dendrite; s, spine; SM, soma. Scale bars: **A,C,D**, 0.5 μm; **B,E‐I**, 0.2 μm.

**Figure 5:**
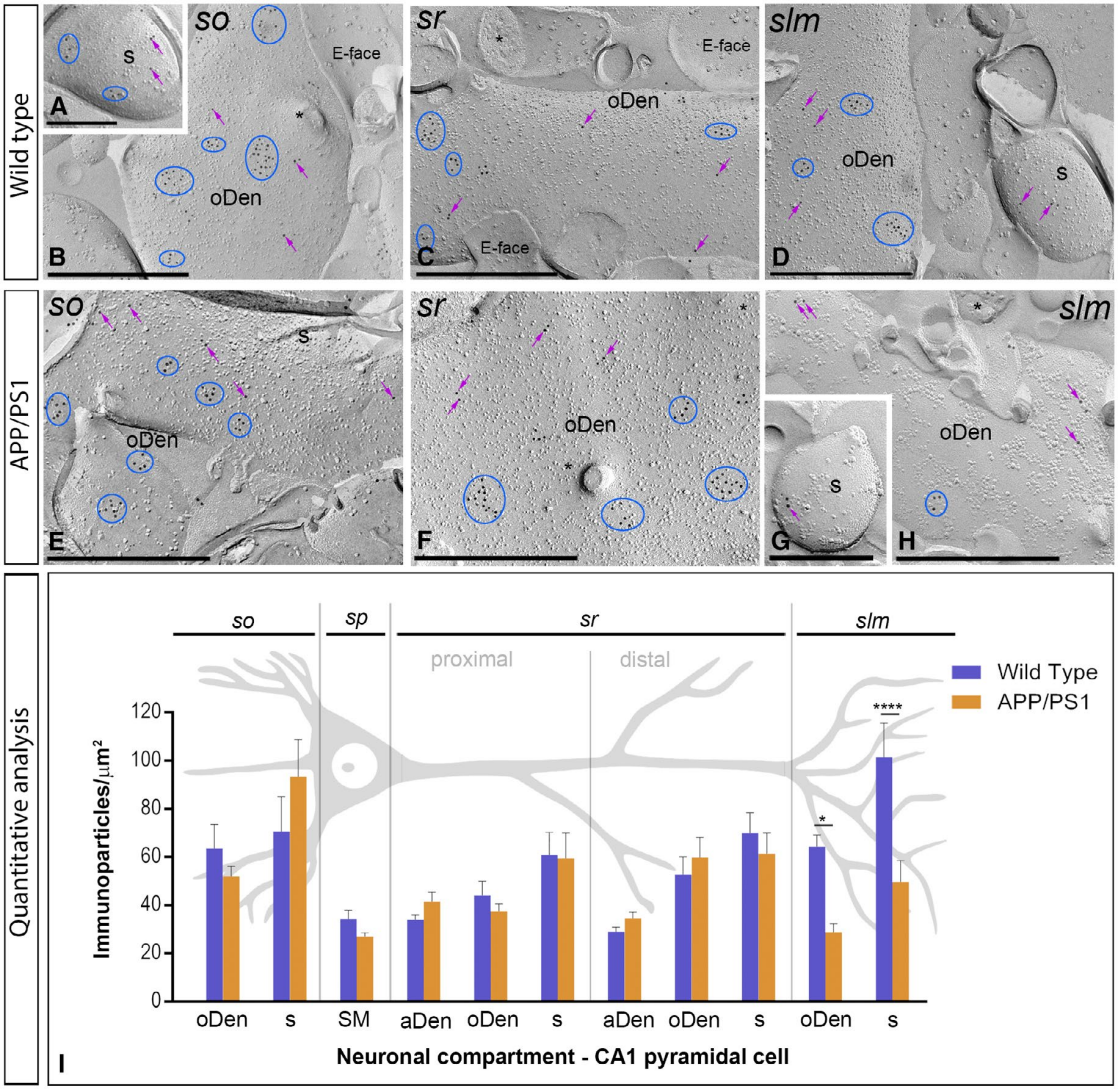
*Subcellular localization of GABA_B1_ in the hippocampus in wild‐type and APP/PS1 mice at 6 months*. Electron micrographs obtained from different strata of the CA1 region showing immunoparticles for GABA_B1_ along the membrane surface of pyramidal cells, as detected using the SDS‐FRL technique. **A‐H**. In wild type and APP/PS1, clusters of GABA_B1_ immunoparticles (blue ellipses/circles) associated with the P‐face were detected in dendritic shafts, illustrated here for oblique dendrites (oDen) and dendritic spines (s) of pyramidal cells in all strata of the CA1 region. Lower density of immunoparticles for GABA_B1_ was also detected scattered (purple arrows) outside the clusters. Fractured spine necks and other cross‐fractures are indicated with asterisks (*). The E‐face is free of any immunolabeling. **I**. Quantitative analysis of GABA_B1_ immunogold labeling in 11 neuronal compartments of CA1 pyramidal cells. The density gradient of membrane surface GABA_B1_ immunoparticles was very similar between wild‐type and APP/PS1 mice in all strata except in the *stratum lacunosum‐moleculare* (slm), where we found significant differences in both oblique dendrites (WT: 60.27 ± 5.60 immunoparticles/µm^2^; APP/PS1: 35.37 ± 3.61 immunoparticles/µm^2^) and dendritic spines (WT: 103.68 ± 18.69 immunoparticles/µm^2^; APP/PS1: 58.09 ± 13.78 immunoparticles/µm^2^) (Two‐way ANOVA test and Bonferroni *post hoc* test, *P < 0.05; ****P < 0.0001). Error bars indicate SEM. Abbreviations: *so*, *stratum oriens*; *sp*, *stratum pyramidale*; *sr*, *stratum radiatum*; *slm*, *stratum lacunosum‐moleculare*; aDen, apical dendrite; oDen, oblique dendrite; s, spine; SM, soma. Scale bars: **A,G**, 0.25 μm; **B‐F,H**, 0.5 μm.

**Figure 6:**
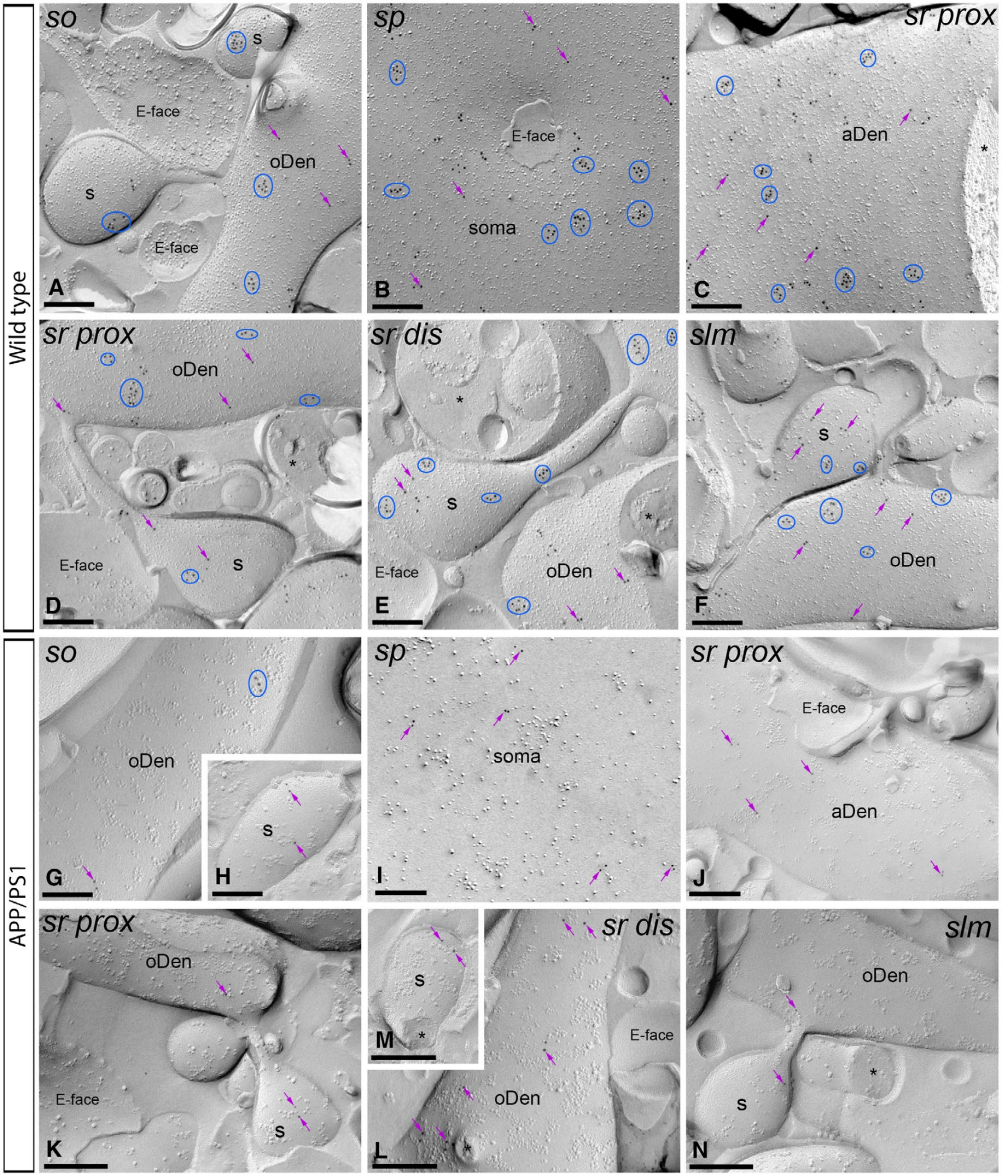
*The density of plasma membrane of GABA_B1_ is reduced in the hippocampus of APP/PS1 mice at 12 months*. Electron micrographs of the CA1 region showing immunoparticles for GABA_B1_ along the membrane surface of pyramidal cells, as detected using the SDS‐FRL technique. **A‐F**. In wild type, clusters of GABA_B1_ immunoparticles (blue ellipses/circles) associated with the P‐face were detected in dendritic shafts, illustrated here for oblique dendrites (oDen) and dendritic spines (s) of pyramidal cells in all strata of the CA1 region. Lower density of immunoparticles for GABA_B1_ was also detected scattered (purple arrows) outside the clusters. **G‐N**. In APP/PS1, surface GABA_B1_ immunoparticles were detected in the P‐face of all compartments of CA1 pyramidal cells. However, GABA_B1_ immunoparticles were detected at lower frequency forming clusters (blue ellipses/circles), being showing a scattered distribution (purple arrows). Fractured spine necks and other cross‐fractures are indicated with asterisks (*). The E‐face is free of any immunolabeling. Scale bars: **A‐N**, 0.2 μm.

**Figure 7:**
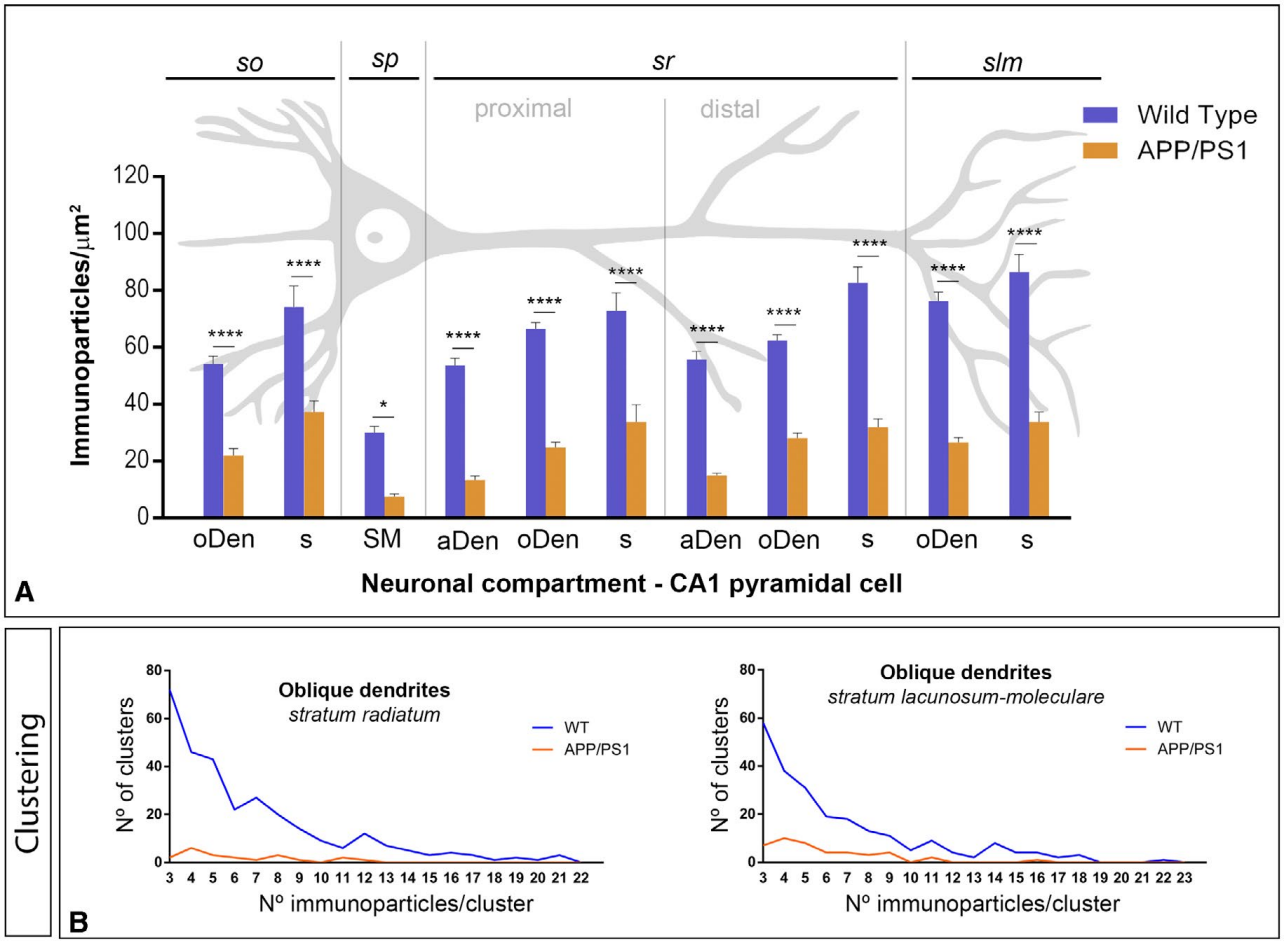
*Density gradient of GABA_B1_ immunoparticles along the* membrane surface *of CA1 pyramidal cells in wild type and APP/PS1 mice at 12 months*. **A**. Quantitative analysis of GABA_B1_ immunogold labeling in eleven neuronal compartments of CA1 pyramidal cells. The density gradient of membrane surface GABA_B1_ immunoparticles was significantly reduced in the APP/PS1 mice compared to age‐matched wild type controls in all strata and compartments analyzed Two‐way ANOVA test and Bonferroni *post hoc* test, *P < 0.05; ****P < 0.0001). Error bars indicate SEM. Abbreviations: *so*, *stratum oriens*; *sp*, *stratum pyramidale*; *sr*, *stratum radiatum*; *slm*, *stratum lacunosum‐moleculare*; aDen, apical dendrite; oDen, oblique dendrite; s, spine; SM, soma. **B**. Quantitative analysis showing composition of clusters of GABA_B1_ immunoparticles along the membrane surface in the *strata radiatum* and *lacunosum‐moleculare* in wild‐type and APP/PS1 mice at 12 months on age in oblique dendrites. In the two dendritic layers analyzed, the number and size of clusters of GABA_B1_ immunoparticles was reduced in the APP/PS1 mice (68 clusters with a range of 3 to 12 immunoparticles in the *sr*; 60 clusters with a range of 3 to 13 immunoparticles in the *slm*) compared to age‐matched wild type controls (298 clusters with a range of 3 to 21 immunoparticles in the *sr*; 230 clusters with a range of 3 to 22 immunoparticles in the *slm*).

To explore how GABA_B_ receptors are organized along the plasma membrane of pyramidal cells, as well as their possible alteration in the hippocampus of APP/PS1 mice, the subcellular localization of the receptors was investigated in the CA1 region of hippocampal sections obtained from 1, 6 and 12 months of age wild‐type and APP/PS1 mice. We performed highly‐sensitive SDS‐FRL labeling for GABA_B1_ 33 to accurately visualize the two‐dimensional distribution and precisely calculate the membrane surface densities of this subunit. Consistent with previous pre‐embedding immunogold labelling studies 27, 32, GABA_B1_ was detected at high densities on the postsynaptic membrane of the entire somato‐dendritic compartment of CA1 principal cells (Figures 4, 5, 6, 7).

We initially performed the ultrastructural analysis at 1 month of age (Figure 4A‐G), when no sign of pathology is observed in the hippocampus of APP/PS1 mice. Both in wild‐type and APP/PS1 mice, immunoparticles for GABA_B1_ were detected in dendritic spines, dendritic shafts and somata (Figure 4A‐I). In all neuronal compartments of CA1 pyramidal cells, immunoparticles for GABA_B1_ were mostly found forming clusters, defined as an aggregation of more than three gold particles and less frequently scattered or isolated single gold particle (Figure 4A‐I). The subcellular distribution pattern and intensity of GABA_B1_ labeling observed in wild type at 1 month of age (Figure 4A‐E) was similar to that found in APP/PS1 at the same age (Figure 4F‐I). Virtually no labeling was observed on the E‐face or on cross‐fractures (Figure 4A‐I). Next, we performed a quantitative comparison of the GABA_B1_ densities in 11 different somato‐dendritic compartments in the CA1 region (Figure 4J). Our data revealed a similar graded increase in the density of GABA_B1_ immunoparticles from the soma to the dendritic spines, but more importantly demonstrated similar GABA_B1_ densities along CA1 pyramidal cells in wild‐type and APP/PS1 mice (Figure 4J). Altogether, our data show that the membrane surface localization of GABA_B1_ did not change in 1 month old APP/PS1 mice compared to wild type controls.

Next, we performed the ultrastructural analysis at 6 months of age (Figure 5A‐H), when Aβ deposition in APP/PS1 mice is accumulating in the hippocampus. In wild‐type and APP/PS1 mice, immunoparticles for GABA_B1_ were detected in dendritic spines, dendritic shafts and somata, most frequently forming clusters and less frequently showing a scattered isolated distribution (Figure 5A‐H). The subcellular distribution of GABA_B1_ immunogold labeling observed at 6 months of age was similar in wild‐type and APP/PS1 mice (Figure 5A‐H). Virtually no labeling was observed on the E‐faces or on cross‐fractures (Figure 5A‐H). Quantitative comparison of the GABA_B1_ densities along the membrane surface of CA1 pyramidal neurons revealed two main findings: 1) a graded increase in the density of GABA_B1_ immunoparticles from the soma to the dendritic spines, and 2) similar GABA_B1_ densities along CA1 pyramidal cells in wild‐type and APP/PS1 mice, with exception of the *stratum lacunosum‐moleculare*, where the GABA_B1_ density varied substantially between wild‐type and APP/PS1 mice (Figure 5I). Indeed, our analysis demonstrated the labeling intensity was significantly reduced in oblique dendrites (oDen) and spines in APP/PS1 mice (oDen = 28.83 ± 3.45 immunoparticles/μm^2^, n = 16 dendrites; s = 49.76 ± 8.79 immunoparticles/μm^2^, n = 21 spines) compared to age‐match wild‐type mice (oDen = 64.34 ± 4.86 immunoparticles/μm^2^, n = 16 dendrites; s = 101.58 ± 13.93 immunoparticles/μm^2^, n = 21 spines) (Two‐way ANOVA test and Bonferroni *post hoc* test, *P < 0.05; ****P < 0.0001) (Figure 5I). Altogether, our data at 6 months of age shows that the plasma membrane localization of GABA_B1_ in APP/PS1 is selectively altered in distal dendrites and spines located in the *stratum lacunosum‐moleculare*.

### Reduction of GABA_B1_ in the plasma membrane of 12 months old APP/PS1 mice

We further investigated the membrane localization of GABA_B_ receptors in APP/PS1 mice at 12 months of age, when Aβ deposition is large and cognitive deficits and synapse loss are severe (Figure 6A‐N). Immunoparticles for GABA_B1_ were detected along the membrane surface of CA1 pyramidal cells in dendritic compartments and cell bodies in wild‐ type and APP/PS1 mice (Figure 6A‐F). In all strata and neuronal compartments of CA1 pyramidal cells, most immunoparticles for GABA_B1_ were found in clusters, with three or more particles and less frequently scattered single gold particles outside the clusters (Figure 6A‐F). No labeling was observed on the E‐face or on cross‐fractures (Figure 6A‐F). In APP/PS1 mice, GABA_B1_ immunoparticles were observed in all strata in the CA1 region, but their distribution pattern changed significantly. Thus, both fewer immunoparticles and fewer clusters were detected along the membrane surface of CA1 pyramidal cells (Figure 6G‐N).

Next, we performed a quantitative comparison of the GABA_B1_ densities in somato‐dendritic compartments of CA1 pyramidal cells (Figure 7A). This analysis revealed a significant decrease between wild‐type and APP/PS1 mice in all strata analyzed (Figure 7A). Altogether, our data shows that the membrane surface localization of GABA_B1_ was reduced significantly in 12‐month‐old APP/PS1 mice (Two‐way ANOVA test and Bonferroni *post hoc* test, *P < 0.05; ****P < 0.0001).

Finally, to determine how the reduction in GABA_B1_ density is taking place at the membrane surface of CA1 pyramidal cells in 12‐month‐old APP/PS1 mice, we analyzed the composition of clusters of GABA_B1_ immunoparticles in dendritic shafts and spines along the membrane surface in the different hippocampal layers. We analyzed neuronal compartments in all strata, but provide only the example of oDen in the proximal and distal parts of the *stratum radiatum* and the *stratum lacunosum‐moleculare* (Figure 7B). To avoid differences in the size of dendrites present in each EM picture analyzed or dendritic alterations induced by AD, we performed this analysis in a similar total area (140.00 µm^2^) of oDen. In the proximal part of the *stratum radiatum*, we detected a total of 68 clusters with a range of 3 to 12 immunoparticles in APP/PS1 mice, while detected 298 clusters with a range of 3 to 29 immunoparticles for GABA_B1_ in wild‐type mice (Figure 7B). In the distal part of the *stratum radiatum*, we detected a total of 57 clusters with a range of 3 to 12 immunoparticles in APP/PS1 mice, while detected 204 clusters with a range of 3 to 29 immunoparticles for GABA_B1_ in wild‐type mice (Figure 7B). Similarly, in the *stratum lacunosum‐moleculare*, we detected a total of 60 clusters with a range of 3 to 13 immunoparticles in APP/PS1 mice, while we detected 234 clusters with a range of 3 to 37 immunoparticles for GABA_B1_ in wild‐type mice (Figure 7B). Altogether, the analysis showed that the number and the size of clusters of GABA_B1_ immunoparticles were reduced in the two dendritic layers in the APP/PS1 mice.

### Reduction in the density of presynaptic GABA_B1_ in 12‐month‐old APP/PS1 mice

**Figure 8:**
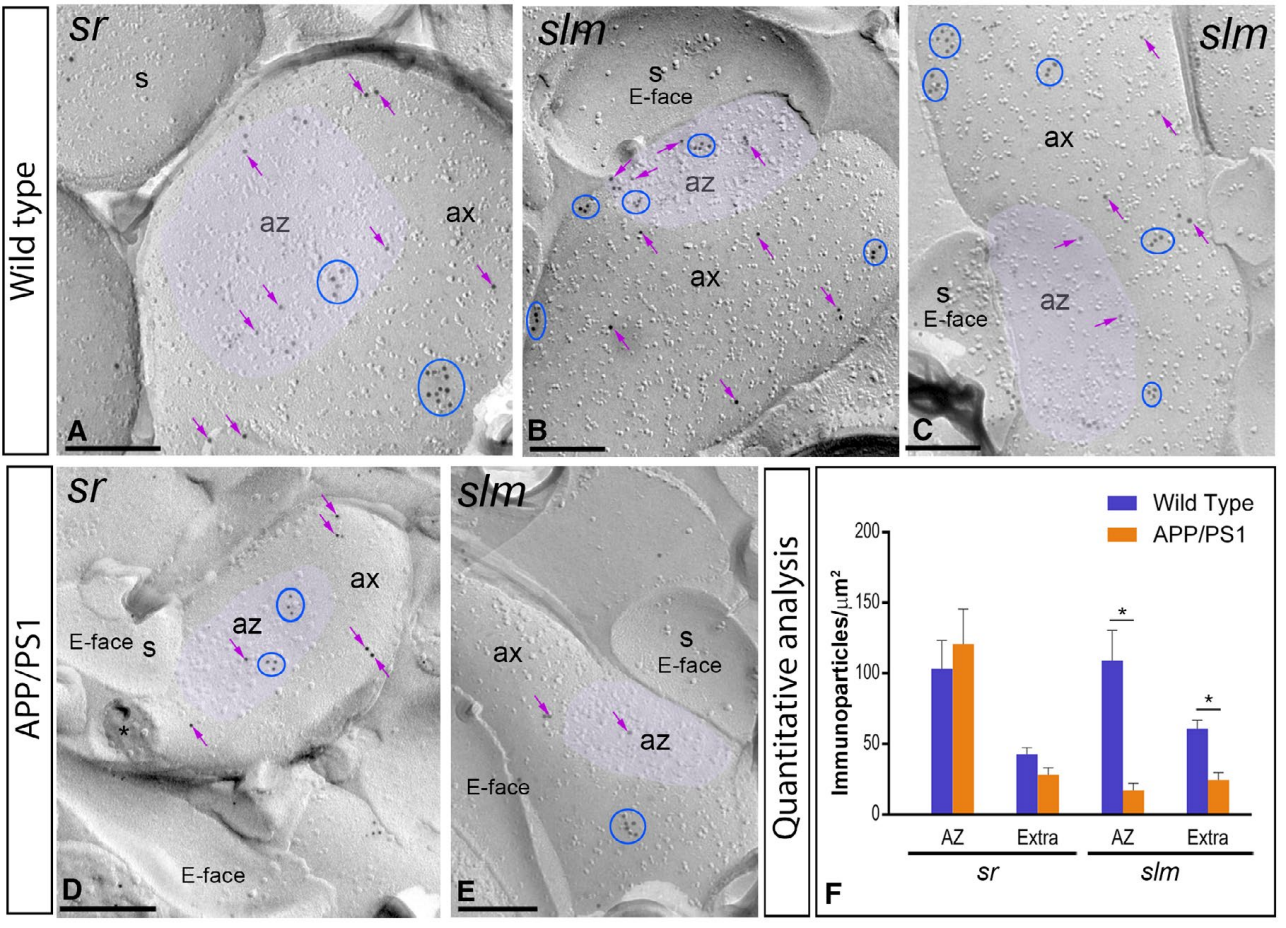
*Presynaptic localization of GABA_B1_ in the hippocampus in wild‐type and APP/PS1 mice*. Electron micrographs showing immunoparticles for GABA_B1_ in presynaptic compartments in the *strata radiatum* (*sr*) and *lacunosum‐moleculare* (*slm*) of the CA1 region of the hippocampus at 12 months of age, as detected using the SDS‐FRL technique. **A‐C**. In wild type, immunoparticles for GABA_B1_ were found within the active zone (az, purple overlay), recognized by the concave shape of the P‐face and the accumulation of IMPs, and along the extrasynaptic site of axon terminals (ax), forming clusters (blue ellipses/circles) and also detected scattered (purple arrows) outside the clusters. **D,E**. In APP/PS1, fewer immunoparticles for GABA_B1_, forming clusters (blue ellipses/circles) or scattered (purple arrows), were detected within the active zone (az, purple overlay) and along the extrasynaptic plasma membrane of axon terminals (ax). Cross‐fractures are indicated with asterisks (*). **F**. Densities of immunoparticles for GABA_B1_ in presynaptic compartments in the *sr* and *slm* in wild‐type and APP/PS1 mice. No differences were detected in densities of GABA_B1_ immunoparticles in the *sr* (WT: AZ = 103.31 ± 20.16 immunoparticles/µm^2^ and Extra = 42.97 ± 4.47 immunoparticles/µm^2^; APP: AZ = 120.95 ± 24.58 immunoparticles/µm^2^; Extra = 28.08 ± 4.96 immunoparticles/µm^2^), but significant differences were detected in the *slm* (WT: AZ = 109.03 ± 21.62 immunoparticles/µm^2^ and Extra = 60.79 ± 6.11 immunoparticles/µm^2^; APP: AZ = 17.32 ± 4.99 immunoparticles/µm^2^; Extra = 24.64 ± 5.17 immunoparticles/µm^2^) (Two‐way ANOVA test and Bonferroni *post hoc* test, *P < 0.05). Error bars indicate SEM. Scale bars: **A‐E**, 0.2 μm.

Immunoparticles for GABA_B1_ were not only confined to somato‐dendritic domains of CA1 pyramidal cells, but also present in axon terminals, as previously reported 27, 32. Thus, we further investigated whether the localization of GABA_B1_ at presynaptic sites is altered in the hippocampus of APP/PS1 (Figure 8), paralleled to the decrease found in somato‐dendritic compartments at 12 months of age. We performed SDS‐FRL labeling for GABA_B1_ to unravel its membrane surface densities in the *strata radiatum* and *lacunosum‐moleculare* of the CA1 region. In wild‐type animals, immunoparticles for GABA_B1_ were detected within the active zone of axon terminals, identified by the concave shape of the P‐face and the accumulation of IMPs 23 and along the extrasynaptic site of axon terminals (Figure 8A‐C). GABA_B1_ immunoparticles were detected both forming clusters and scattered outside the clusters, both in the active zone and at the extrasynaptic sites (Figure 8A‐C). The density of GABA_B1_ immunoparticles was higher at the active zones (AZ; *sr* = 103.31 ± 20.16 immunoparticles/µm^2^, n = 82 particles; *slm* = 109.03 ± 21.62 immunoparticles/µm^2^, n = 149 particles) than at the extrasynaptic sites (Extra; *sr* = 42.97 ± 4.47 immunoparticles/µm^2^, n = 305 particles; *slm* = 60.79 ± 6.11 immunoparticles/µm^2^, n = 347 particles) (Figure 8F). In APP/PS1 animals, GABA_B1_ immunoparticles were detected in the same presynaptic compartments also showing larger density at the active zone terminals than at extrasynaptic sites, showing clusters and scattered distributions (Figure 8D‐E). However, quantitative comparisons with age‐matched wild‐type mice did not show differences in the density of GABA_B1_ immunoparticles in the *sr* (AZ = 120.95 ± 24.58 immunoparticles/µm^2^, n = 79 particles; Extra = 28.08 ± 4.96 immunoparticles/µm^2^, n = 81 particles) (Two‐way ANOVA test and Bonferroni *post hoc* test, p = 0.68), while we found significant differences in the *slm* (AZ = 17.32 ± 4.99 immunoparticles/µm^2^, n = 19 particles; Extra = 24.64 ± 5.17 immunoparticles/µm^2^, n = 67 particles) (Two‐way ANOVA test and Bonferroni *post hoc* test, *P < 0.05) (Figure 8F).

To test the consistency and specificity on the reduction of GABA_B_ receptors in the APP/PS1 mice described above, as control of our technical approach and mouse model we analysed the distribution of four different proteins: (i) AMPA receptors, known to localise in excitatory synapses in an activity‐dependent manner (Supplementary Figure S1); (ii) the GluN1 subunit of NMDA receptors, which are not incorporated in an activity‐dependent manner into synapses (Supplementary Figure S2); (iii) the GIRK2 subunit of G protein‐gated inwardly rectifying potassium channels, which are known effector ion channels of GABA_B_ receptors (Supplementary Figure S2); and (iv) the synaptosomal‐associated protein SNAP‐25, which is responsible for the Ca^2+^‐dependent exocytosis of neurotransmitters, thus playing a key role to normal functioning of brain (Supplementary Figure S2). Thus, using the SDS‐FRL method, we determined the densities of the four proteins receptors at synapses of the *stratum lacunosum‐moleculare* (the subfield in which we detected most alteration in the localization of GABA_B_ receptors) in the CA1 region of hippocampal sections. In APP/PS1 mice, compared to control mice, lower density of immunoparticles for AMPA (Supplementary Figure S1) and NMDA (Supplementary Figure S2) were detected in excitatory synapses of dendritic spines, fewer immunoparticles for GIRK2 (Supplementary Figure S2) were detected in dendritic shafts and spines of pyramidal cells and fewer immunoparticles for SNAP‐25 (Supplementary Figure S2) were detected in axon terminals establishing excitatory synapses with dendritic spines. These reductions, which are in agreement with the current literature, validate the effect described for GABA_B_ receptors.

### Increase of GABA_B1_ in the cytoplasm of CA1 pyramidal cells and axon terminals in APP/PS1 mice

We next sought to investigate why the localization of GABA_B1_ in the membrane surface of CA1 pyramidal cells and axon terminals is significantly reduced in the hippocampus of APP/PS1 at 12 months of age when the total amount of protein remains invariable, as shown by histoblots and western blots. To explore possible receptor internalization and accumulation in intracellular domains of CA1 pyramidal cells and axon terminals of APP/PS1 mice compared to age‐matched wild type controls, the subcellular localization of GABA_B1_ was investigated at 1, 6 and 12 months of age. We used the pre‐embedding immunogold technique and quantitative analysis on tissue blocks taken from the proximal part of the *stratum radiatum* and the *stratum lacunosum‐moleculare* of the CA1 area.

**Figure 9:**
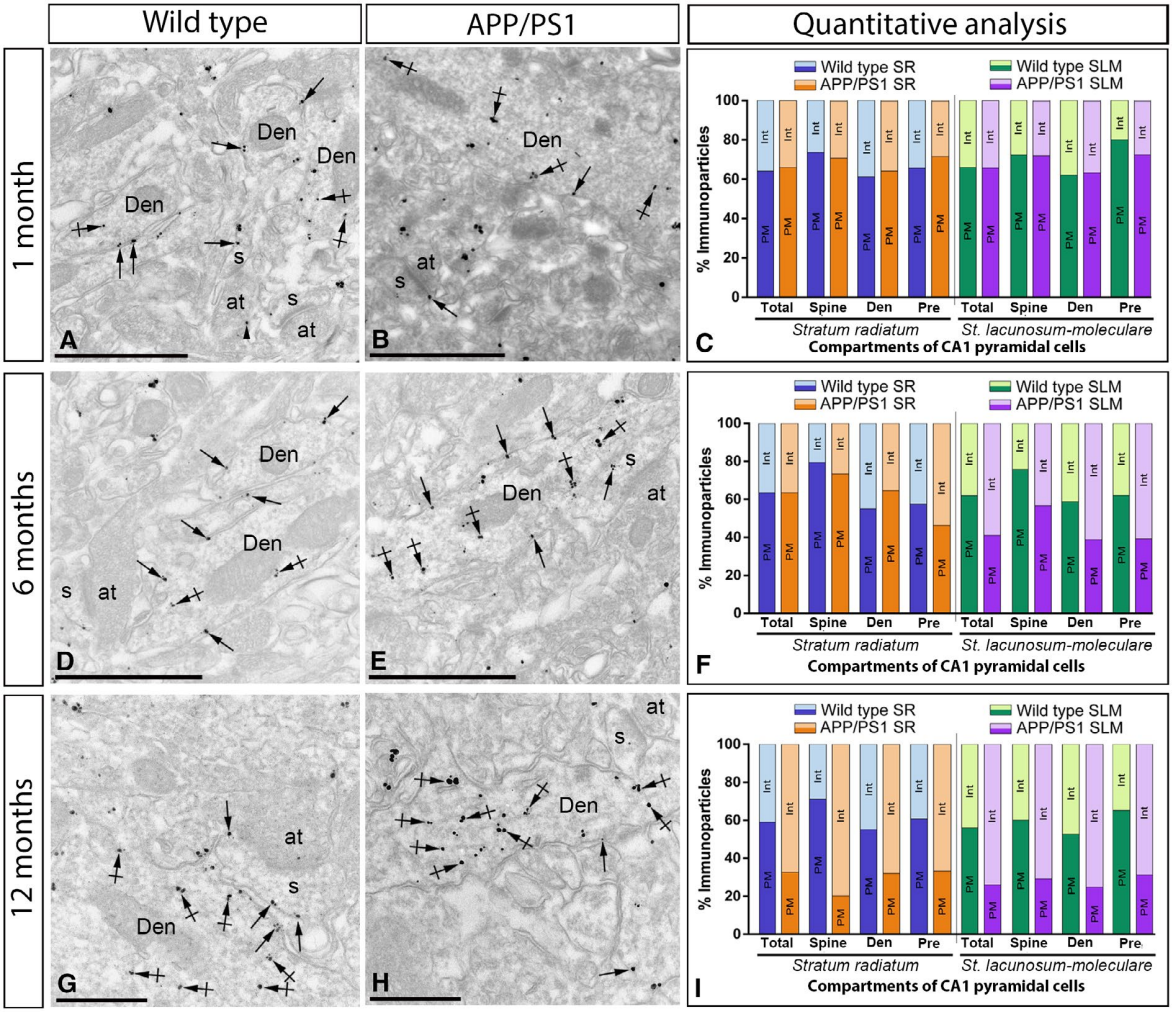
*Intracellular distribution of GABA_B1_ is increased in the hippocampus of APP/PS1 mice*. Electron micrographs showing immunoparticles for GABA_B1_ in pyramidal cells of the CA1 region at 1, 6 and 12 months of age in wild ‐type and APP/PS1 mice, as detected using a pre‐embedding immunogold technique and obtained from the *stratum lacunosum‐moleculare* for illustrative purposes. **A,B,D,E,G,H**. In all those animals and ages, GABA_B1_ immunoparticles were found along the extrasynaptic plasma membrane (arrows) and intracellular sites (crossed arrows) of dendritic shafts (Den) and spines (s) of CA1 pyramidal cells contacted by axon terminals (at), and less frequently at presynaptic sites (arrowheads). **C**. Quantitative analysis at 1 month in the *strata radiatum* and *lacunosum‐moleculare* showed that the proportion of postsynaptic GABA_B1_ immunoparticles along the plasma membrane (65.52% in *sr* and 65.11% in *slm*) and intracellular sites (34.48% in *sr* and 34.89% in *slm*) in APP/PS1 was similar to age‐matched wild‐type mice (Plasma membrane: 64.04% in *sr* and 64.68% in *slm*; Intracellular: 35.96% in *sr* and 35.32% in *slm*) in dendritic spines and shafts of CA1 pyramidal cells. **F**. Quantitative analysis at 6 months showed no changes in the *stratum radiatum* (Plasma membrane: 63.45% in wild type and 63.46% in APP/PS1; Intracellular: 36.55% in wild type and 36.54% in APP/PS1), but a clear reduction of plasma membrane GABA_B1_ at postsynaptic compartments, with a subsequent increase in cytoplasmic sites, in APP/PS1 mice (Plasma membrane: 62.06% in wild type and 41.05% in APP/PS1; Intracellular: 37.94% in wild type and 58.05% in APP/PS1). (I) Quantitative analysis at 12 months of age showing that GABA_B1_ immunoparticles were less frequently observed along the extrasynaptic plasma membrane dendrites and spines of CA1 pyramidal cells in APP/PS1 mice, as well at presynaptic sites, but instead they more frequently detected at intracellular sites in both the *strata radiatum* (Plasma membrane: 58.81% in wild type and 32.60% in APP/PS1; Intracellular: 41.19% in wild type and 67.40% in APP/PS1), proximal and distal parts, and *lacunosum‐moleculare* (Plasma membrane: 54.23% in wild type and 15.19% in APP/PS1; Intracellular: 45.77% in wild type and 84.81% in APP/PS1). Scale bars: A,B,D,E, 1 μm; G,H, 0.5 μm.

At 1 month of age, immunoreactivity for GABA_B1_ was primarily found along the extrasynaptic plasma membrane of dendritic spines and shafts of CA1 pyramidal cells and also associated at intracellular sites in dendritic spines (Figure 9A,B). Quantitative analysis performed in the *strata radiatum (sr)* and *lacunosum‐moleculare (slm)* of the CA1 area showed similar proportion of postsynaptic GABA_B1_ immunoparticles in the plasma membrane vs. intracellular in APP/PS1 (Plasma membrane: 65.52% in *sr* and 65.11% in *slm*; Intracellular: 34.48% in *sr* and 34.89% in *slm*) and age‐matched wild‐type mice (Plasma membrane: 64.04% in *sr* and 64.68% in *slm*; Intracellular: 35.96% in *sr* and 35.32% in *slm*) (Figure 9C).

At 6 months of age, immunoreactivity for GABA_B1_ followed similar distribution than at 1 month, being also observed along the extrasynaptic plasma membrane and intracellular sites of dendritic spines and shafts of CA1 pyramidal cells (Figure 9D‐E). However, quantitative analysis showed some differences in the plasma membrane vs. intracellular between the *stratum radiatum* and the *stratum lacunosum‐moleculare* of the CA1 area. Thus, in the *stratum radiatum* we did not detect differences in the total amount of postsynaptic immunoparticles between wild‐type and APP/PS1 mice (Plasma membrane: 63.45% in wild type and 63.46% in APP/PS1; Intracellular: 36.55% in wild type and 36.54% in APP/PS1) (Figure 9F). Consistent with this data, similar percentage of immunoparticles were observed in dendritic spines and shafts between wild‐type and APP/PS1 mice (Figure 9F). However, we detected changes in the *stratum lacunosum‐moleculare* in the plasma membrane vs. intracellular among wild‐type and APP/PS1 mice (Plasma membrane: 62.06% in wild type and 41.05% in APP/PS1; Intracellular: 37.94% in wild type and 58.05% in APP/PS1) (Figure 9F).

At 12 months of age, immunoreactivity for GABA_B1_ was again localized along the extrasynaptic plasma membrane of dendritic spines and shafts, as well as associated at intracellular sites in dendritic spines (Figure 9G‐I). Quantitative analysis showed clear differences in the plasma membrane vs. intracellular both in the *stratum radiatum* (Plasma membrane: 58.81% in wild type and 32.60% in APP/PS1; Intracellular: 41.19% in wild type and 67.40% in APP/PS1) and the *stratum lacunosum‐moleculare* (Plasma membrane: 54.23% in wild type and 15.19% in APP/PS1; Intracellular: 45.77% in wild type and 84.81% in APP/PS1) of the CA1 area. These differences in plasma membrane vs. intracellular were detected in dendritic shafts and spines in the two strata (Figure 9G‐I). To clarify the intracellular compartments on which GABA_B1_ is accumulated, we performed double labeling immunofluorescence with different markers (Rab4, EEA1, LAMP1 or CHOP). We found no differences in the co‐localization pattern in the APP/PS1 mice compared to age‐matched wild type controls with the makers employed (Supplementary Figure S4).

Presynaptically, immunoreactivity for GABA_B1_ was occasionally detected in axon terminals establishing asymmetrical synapses with dendritic spines (Figure 9). At 1 month of age, GABA_B1_ presynaptic labeling was similar between wild‐type and APP/PS1 animals (Figure 9C). At 6 months of age, we detected changes in the plasma membrane vs. intracellular among wild type and APP/PS1 in the *stratum radiatum* (Plasma membrane: 57.57% in wild type and 46.30% in APP/PS1; Intracellular: 42.42% in wild type and 53.70% in APP/PS1) and *stratum lacunosum‐moleculare* (Plasma membrane: 62.12% in wild type and 39.25% in APP/PS1; Intracellular: 37.88% in wild type and 60.75% in APP/PS1) (Figure 9F). These differences were more pronounced at 12 months of age, detecting changes in the *stratum radiatum* (Plasma membrane: 60.78% in wild type and 33.33% in APP/PS1; Intracellular: 39.22% in wild type and 66.67% in APP/PS1) and *stratum lacunosum‐moleculare* (Plasma membrane: 65.22% in wild type and 31.25% in APP/PS1; Intracellular: 34.78% in wild type and 68.75% in APP/PS1) (Figure 9I).

Finally, to validate the described reduction of post and presynaptic GABA_B_ receptors in the hippocampus of APP/PS1 mice, we determined their expression levels together with other markers in human tissue. Thus, using immunoblots we assessed the expression levels of GABA_B1_, GluA1, GluA2 and GluN1 in tissue lysates of the hippocampus in control and AD cases (Supplementary Figure S3A). Quantification of all these immunoreactive bands revealed that GABA_B1_, GluA1, GluA2 and GluN1 proteins were significantly reduced in the hippocampus of AD subjects compared to controls (Supplementary Figure S3B).

## Discussion

To understand the mechanisms that are operative in AD, it is important to appreciate that alteration of neurotransmitter receptors must be a common denominator of dendritic spines loss and synaptic impairment, two critical events in the pathophysiology of this neurodegenerative disease 42, 48, 54. We hypothesize that neurons experiencing AD neuropathology may selectively regulate neurotransmitter receptors associated with excitatory synapses, thus contributing to neuronal damage and to the cognitive decline associated with AD. However, the receptors and ion channels involved in the synaptic deficits present in pathological conditions are still poorly known. Identification of the molecular mechanisms at central synapses affected by AD may allow developing more effective therapeutic approaches for AD, for which no curative treatment is yet available. Here, we have demonstrated for the first time a compartment‐ and age‐dependent reduction of surface GABA_B_ receptors and a parallel intracellular increase of these receptors in CA1 pyramidal cells with progressive neuropathology in an AD mouse model, the APP/PS1 model. We additionally show that the decrease of GABA_B_ receptors also affects in an age‐dependent manner axon terminals contacting CA1 pyramidal cells. The significant reduction in GABA_B_ receptors may contribute to the pathology and memory impairment associated with AD.

### Unchanged expression of hippocampal GABA_B_ receptor protein in Alzheimer's disease

Dysfunction of the hippocampus largely contributes to the memory deficits that characterise AD 31, 44. In contrast to the large interest focused on the potential role of glutamate receptors in AD 11, 39, less information is available about the involvement of GABA receptors. In particular, GABA_B_ receptors also play a role in learning and memory 19. Recent studies reported that GABA_B_ receptors selectively co‐purify with APP 47 and this macromolecular complex links presynaptic GABA_B_ receptor trafficking to Aβ formation 9. In addition, GABA_B1a_ has been identified as a receptor for secreted APP, which would regulate the function of the receptor isoform to modulate synaptic transmission and plasticity 45. For these reasons, our work adopted transgenic mice overexpressing mutant familial AD genes [amyloid‐β protein precursor (AβPP), presenilin‐1 (PS1), and PS2], considered one of the most relevant animal models of AD. These animals show Aβ deposition by 4 months with a progressive increase in senile plaque number up to 12 months and cognitive impairments 14, 15.

In the present study, we demonstrated with different approaches that total protein levels of GABA_B_ receptors were not significantly changed in APP/PS1 mice with the progressive increase of Aβ levels at different ages in the hippocampus. This does not seems to be the case for other components of the GABA receptor systems, as GABA_A_ receptors are differentially affected in AD in a region‐ and subunit‐dependent manner. Thus, some GABA_A_ subunits showed decrease expression in the human hippocampus, although some others remain unchanged 28. In contrast, our data also show reduction of GABA_B1_ protein level in the hippocampus of AD patients, consistent with the reported downregulation in the gene expression of GABA_B1_ in AD 43. This discrepancy could be due to posttranscriptional changes in the control of gene expression at the RNA level, but more likely could be the result of differences in brain organisation between humans and mice, because the APP/PS1 model does not develop neurofibrillary tangles, other typical pathologic alterations observed in AD human patients, or because the stages of AD in the patients were more severe than the AD neuropathology suffered by mice at 12 months of age.

Ultrastructural studies in the hippocampus have shown that GABA_B_ receptors are enriched in the vicinity of excitatory synapses, supporting the idea that glutamate receptors can influence GABA_B_ receptor signaling 27, 32, 53. Indeed, NMDA receptor activation can induce phosphorylation on the GABA_B1_ and GABA_B2_ subunits to promote endocytosis 17, 51. GABA_B_ receptors can also influence the NMDA‐mediated excitatory postsynaptic potentials in the CA1 region of the hippocampus 38. An autoradiography study of GABA_B_ receptor expression in the human hippocampus of AD patients indicated fewer GABA_B_ receptors in the molecular layer of the dentate gyrus, and the *strata pyramidale* and *lacunosum‐moleculare* of the CA1 region 7. Our current knowledge of the complexity of receptor organization in central neurons demands more exacting techniques that allow precise identification and localization of individual receptor subtypes to unravel their distribution in AD. Thus, the SDS‐FRL and pre‐embedding immunogold techniques were the methods of choice to decipher the density of GABA_B1_ in hippocampal neurons.

### Plasma membrane reduction of GABA_B_ receptors in the hippocampus of Alzheimer's disease

One of the major research aims in this work has been to examine the possibility that, despite the unchanged protein levels in the APP/PS1 model of AD, GABA_B_ receptors may still undergo changes in their localization along the plasma membrane vs. cytoplasmic sites with pathology progression. For that purpose we concentrated in the CA1 region of the hippocampus using the SDS‐FRL technology, proved to be an excellent tool to study the high‐resolution subcellular localization of surface‐localized proteins 50. Using this technique, we were able to fully map the two‐dimensional distribution of GABA_B_ receptors obtaining very accurate data about their density in different compartments of CA1 pyramidal cells in brains displaying AD pathology at a level of detail never previously attained.

Our analysis revealing that GABA_B_ receptors are differentially localized to somato‐dendritic compartments of hippocampal principal cells, both in wild type and APP/PS1 at the three ages studied is in line with results of previous immunoEM investigations performed in young adult animals 12, 27, 32, 53. More importantly, the principal and exciting finding from our results is the reduction in the density of plasma membrane‐targeted GABA_B_ receptors in an age‐ and compartment‐dependent manner. Thus, we detected significant differences in the plasma membrane distribution of GABA_B1_ and subsequent increase at cytoplasmic sites only in the *stratum lacunosum‐moleculare*, but found no evidence for changes in all other pyramidal cell compartments, at 6 months of age. This observation is consistent with the evidence that density of binding sites for GABA_B_ receptors are decreased in *stratum lacunosum‐moleculare* of the CA1 region in human hippocampus with AD 7. Since this hippocampal region is the common location for large accumulation of Aβ in AD 22, the decrease in density of surface GABA_B1_ in the *stratum lacunosum‐moleculare* of the CA1 region may reflect changes due to entorhinal cortical pathology. Previous work demonstrated that AD pathology begins in the entorhinal cortex and spreads through synaptic connections to the hippocampus 31 and the resulting hippocampal synaptic weakening induced in the entorhinal cortex is a consequence of the pathologic increase of Aβ and p‐tau 49.

At more advanced stages of AD pathology APP/PS1 mice show high‐density of Aβ and severe cognitive impairments develop 2, 15. Our analysis performed at 12 months of age revealed a significant reduction in the membrane surface distribution of GABA_B1_ in all neuronal compartments. The possibility that these immunohistochemical findings in APP/PS1 are due to technical issues or storage conditions is unlikely because wild‐type and APP/PS1 mice were always matched from same litters and although dystrophic dendrites were observed in these transgenic mice 2 our investigation was performed in the Aβ plaque‐free regions from these animals. Following the finding on the reduction of GABA_B1_ along the membrane surface of CA1 pyramidal cells in APP/PS1 mice, we investigated potential internalization at cytoplasmic sites. Immunoelectron microscopy and quantitative analysis showed that there is an accumulation of immunoparticles for GABA_B1_ at cytoplasmic sites. Given that the total amount of GABA_B1_ protein does not change between wild‐type and APP/PS1 mice, our data suggests that the internalized pool of GABA_B_ receptors was not redirected to subsequent lysosomal degradation in AD, in contrast to the activity‐dependent degradation of GABA_B_ receptors observed in hippocampal and cortical neurons 17, 51. Similar plasma membrane‐cytoplasmic redistribution of GABA_B_ receptors has been reported in GABAergic neurons of the VTA in methamphetamine‐injected mice 39.

An additional, interesting finding of the present study is that reduction of membrane surface GABA_B1_ in our model of AD was due to alteration in its clustering, with lower number of clusters and fewer immunoparticles per cluster. GABA_B_ receptors form macromolecular signaling complexes with GIRK channels and Cav channels 40. Although no information is yet available about the alterations of these effector ion channels in AD, the decrease of GABA_B_ receptors from the membrane surface of CA1 pyramidal cells, together with the decrease of GIRK2 channels we detected in the same compartments, can produce alteration in GABA_B_‐mediated GIRK currents 39 and this could involve a change in G protein‐coupling efficiency with effector ion channels 29 or simply that fewer receptors can couple to channels. Altogether, this alteration might be enough to reduce GIRK and/or Cav channel conductance in CA1 pyramidal neurons, thus playing a potential role in the hippocampal activity dysfunction observed in AD, although this needs to be confirmed with electrophysiology.

On the presynaptic side, GABA_B_ receptors mediate slow and prolonged synaptic inhibition by activating Cav channels, thus modulating the release of neurotransmitters 3. In the hippocampus, synaptically released GABA can inhibit excitatory neurotransmission at the Schaffer collateral synapses in CA1 acting through presynaptic GABA_B_ receptors 24. In this study we observed immunoparticles for GABA_B1_ along the extrasynaptic membrane and the active zone or presynaptic membrane specialization of axon terminals in different layers of the CA1 region, in line with previous reports using different ultrastructural approaches 27, 53. Similarly to the decrease of postsynaptic GABA_B_ receptors, we detected a reduction of presynaptic GABA_B1_ in APP/PS1 mice of 12 months and this reduction was more pronounced in the *stratum lacunosum‐moleculare*. This data are in agreement with a recent study reporting that the increase in Aβ production is linked to dysfunctional axonal trafficking and reduced GABA_B_ receptor expression in the APP‐/‐ mice 9 and also consistent with the study showing that secreted APP binds to GABA_B1a_ 45, an isoform preferentially localized at presynaptic sites 53, to supress synaptic vesicle release and thus modulating synaptic transmission and plasticity. One way to reduce or supress the release of neurotransmitter from synaptic vesicles is by decreasing the number of involved receptors in the presynaptic plasma membrane, consistent with the reduction of GABA_B_ receptors we have reported at presynaptic sites.

## Conclusions

Our work explored the pre and postsynaptic alteration of GABA_B_ receptors in hippocampal CA1 pyramidal cells in a mouse model of AD, providing new insights into neurotransmitter receptor redistribution at different stages of AD pathology. This work shows a severe reduction of membrane surface GABA_B_ receptors and subsequent accumulation at cytoplasmic sites only at those ages and in those neuronal compartments suffering a decrease from the plasma membrane. The mechanisms behind these changes in subcellular distribution and their functional implications are still not known. However, the present study and future data in human tissue are important to explore how GABA_B_ receptors are involved in the altered neuronal excitability associated with AD, essential information for the development of therapeutic strategies for disease improvement. One way of therapeutic intervention would be stabilizing of GABA_B_ receptors in the neuronal surface, controlled by heteromerization with other proteins, and by phosphorylation and dephosphorylation events, and regulated by changes in glutamatergic excitatory activity.

## Conflict of Interest

The authors of this manuscript declare that they have no conflict of interests.

## Author Contributions

All authors had full access to all data in the study and take responsibility for the integrity of the data and the accuracy of the data analysis. RL and YF designed the project; AMB, RL and YF performed SDS‐ FRL immunoelectron microscopy; AMB, CA and AEMM performed histoblot analysis; AMB performed western blots; AMB, RAR and AEMM performed light microscopy immunohistochemistry; LDLO developed in‐house software and performed computational analysis; RS provided reagents and feedback on the quantitative analysis; JMH and AB provided APP/PS1 and WT tissues and feedback on the manuscript; SF and BB provided knock‐out tissues and feedback on the manuscript; AMB, RAR, CA and RL analysed data; RL wrote the paper. All authors read and approved the final manuscript.

## Consent for Publication

All co‐authors of the present manuscript can certify that it has not been submitted to more than one journal for simultaneous consideration and that the manuscript has not been published previously (partly or in full). The authors also can certify that our main study is not split up into several parts to increase the quantity of submissions, that none of the data presented here have been fabricated or manipulated and that we present our own data/text/theories/ideas. All authors and authorities have explicitly provided their consent to submit the present manuscript and in general we all agree with the ethical responsibilities of authors of the journal. Finally, all authors give consent for publication in *Brain Pathology*.

## Ethical Approval and Consent to Participate

All animal experimental procedures were performed in accordance with Spanish (RD 1201/2015) and European Union regulations (86/609/EC) and the protocols were approved by the local Animal Care and Use Committee.

## Supporting information


**Figure S1**. *Reduced density of synaptic AMPA receptors in dendritic spines of APP/PS1 mice at 12 months*. (**A‐F**) Electron micrographs of the hippocampus showing immunoparticles for pan‐AMPA at excitatory synaptic sites of dendritic spines of pyramidal cells in the CA1 *stratum lacunosum‐moleculare*, as detected using the SDS‐FRL technique at 1, 6 and 12 months of age. Postsynaptic membrane specializations (IMP clusters, pseudo coloured in blue for wild type and in red for APP/PS1 to aid visualization) show strong immunoreactivity for pan‐AMPA (10 nm gold particles) in the wild type, while they show weaker immunoreactivity in the APP/PS1. Scale bars: **A‐F**, 200 nm. (**G,H,I**) Histograms showing the distribution of densities of gold particles that label AMPA receptors in SDS‐FRL replicas of individual postsynaptic membrane specializations in wild type and APP/PS1 mice. Quantitative analysis showed no changes in the densities of AMPA immunoparticles in excitatory synapses in dendritic spines at 1 and 6 months of age (panels **G** and **H**). However, the analysis showed a significant reduction in the APP/PS1 mice (205.03 ± 16.88 immunoparticles/µm^2^) compared to age matched wild type (423.20 ± 20.33 immunoparticles/µm^2^; Kruskal–Wallis test, pairwise Mann–Whitney U test and Dunn’s method, ***P < 0.001) at 12 months of age. Error bars indicate SEM.
**Figure S2**. *Reduced density of synaptic markers in APP/PS1 mice at 12 months*. (**A‐F**) Electron micrographs of the hippocampus showing immunoparticles for the GluN1 subunit of the NMDA receptors, the GIRK2 subunit of GIRK channels and SNP‐25 in the CA1 *stratum lacunosum‐moleculare*, as detected using the SDS‐FRL technique at 12 months of age. (**A,D**) Strong immunoreactivity for GluN1 (10 nm gold particles) were observed in postsynaptic membrane specializations in the wild type, while weaker immunoreactivity was detected in the APP/PS1. (**G**) Quantitative analysis showing a significant reduction of in the APP/PS1 mice (68.74 ± 11.15 immunoparticles/µm2) compared to age matched wild type (198.56 ± 15.24 immunoparticles/µm2; Kruskal–Wallis test, pairwise Mann–Whitney U test and Dunn’s method, ***p<0.0001). (**B,E**) Immunoparticles for GIRK2 were observed along the surface membrane of dendrites and spines. (**H**) Quantitative analysis showing that immunoreactivity for GIRK2 was significantly reduced in oblique dendrites (oDen) and spines in APP/PS1 mice (oDen = 92.74 ± 18.12 immunoparticles/μm^2^, n = 10 dendrites; s = 128.00 ± 12.38 immunoparticles/μm^2^, n = 9 spines) compared to age‐match wild type mice oDen = 273.10 ± 25.92 immunoparticles/μm^2^, n = 10 dendrites; s = 461.92 ± 59.88 immunoparticles/μm^2^, n = 10 spines) (Two‐way ANOVA test, *P < 0.05; ***P < 0.01). (**C,F**) Immunoreactivity for SNAP‐25 were detected along the extrasynaptic plasma membrane of axon terminals (at) facing dendritic spines (s). (**I**) Our quantitative analysis showed a significant reduction in the density of SNAP‐25 in the APP/PS1 mice (30.73 ± 7.58 immunoparticles/µm^2^) compared to age matched wild type (mean = 134.02 ± 7.46 immunoparticles/µm^2^; t test using Holm‐Sidak method, ***P <0. 001). Scale bars: **A‐F**, 200 nm. Error bars indicate SEM.
**Figure S3**. *Co‐localization of GABA_B_ receptors with marker proteins* *in APP/PS1 mice at 12 months*. (**A‐H**) Immunofluorescence for GABA_B1_ (green) and Rab4 (red), EEA1 (red), LAMP1 (red) or CHOP (red) in wild type and APP/PS1 mice at 12 months of age. The overlay between GABA_B1_ and each marker protein can be seen in yellow (A3‐G3, B3‐H3). Co‐localization between GABA_B1_ and the different marker proteins was detected mainly in the somata of pyramidal cells in the *stratum pyramidale*, but no differences in the frequency of co‐localization was observed in the APP/PS1 mice compared to age‐matched controls. so, *stratum oriens*; sr, *stratum radiatum*. Scale bars: **A‐H**, 20 µm.
**Figure S4**. *Representative western blots of different molecules in the hippocampus from control and Alzheimer´s patients*. (**A**) Crude membrane preparations were subjected to 7.5% SDS‐PAGE, transferred on to polyvinylidene difluoride membranes. They were reacted with an anti‐pan GABA_B1_ antibody, which recognised both GABA_B1a_ and GABA_B1b_ subunits with estimated molecular masses of 130 and 100 kDa, respectively. The antibodies to GluA1, GluA2 detected a single predominant band at 100 kDa and NMDA detected a single predominant band at 120 kDs. (**B**) The developed immunoblots were scanned and densitometric measurements were averaged together to compare the protein densities between controls and AD in the hippocampus. Quantification of GABA_B1_, GluA1, GluA2 and GluN1 normalised to α‐tubulin and expressed as pixel density showed a significant reduction in the amount of proteins when compared AD with controls. An antibody anti α‐Tubulin served as an internal control to ensure comparable protein loading showed no significant difference among AD or control groups. Data are means ± SEM of represented cases. *P  <0. 05; **P < 0.01; ***P < 0.001.
**Figure S5**. *The density of CA1 pyramidal cells is not altered* *in APP/PS1 mice at 12 months*. (**A**) Hippocampal sections from WT and APP/PS1 were stained with DAPI. (**B**) Quantification of the number of nuclei in the pyramidal cell layer of the CA1 region. We confirmed that the number of neurons is similar between WT and APP/PS1. Scale bars: **A‐B**, 100 µm.

## Data Availability

All data used and/or analyzed during the current study were available from the corresponding author on reasonable request.
