# Supplementary material for: Reduction in the neuronal surface of post and presynaptic GABAB receptors in the hippocampus in a mouse model of Alzheimer's disease
Source: Brain Pathol. 2019 Dec 12;30(3):554–75. doi: 10.1111/bpa.12802 (PMC7317930; doi:10.1111/bpa.12802)
Supplement: Supplementary file 1 — Figure S1. Reduced density of synaptic AMPA receptors in dendritic spines of APP/PS1 mice at 12 months. (A‐F) Electron micrographs of the hippocampus showing immunoparticles for pan‐AMPA at excitatory synaptic sites of dendritic spines of pyramidal cells in the CA1 stratum lacunosum‐moleculare, as detected using the SDS‐FRL technique at 1, 6 and 12 months of age. Postsynaptic membrane specializations (IMP clusters, pseudo coloured in blue for wild type and in red for APP/PS1 to aid visualization) show strong immunoreactivity for pan‐AMPA (10 nm gold particles) in the wild type, while they show weaker immunoreactivity in the APP/PS1. Scale bars: A‐F, 200 nm. (G,H,I) Histograms showing the distribution of densities of gold particles that label AMPA receptors in SDS‐FRL replicas of individual postsynaptic membrane specializations in wild type and APP/PS1 mice. Quantitative analysis showed no changes in the densities of AMPA immunoparticles in excitatory synapses in dendritic spines at 1 and 6 months of age (panels G and H). However, the analysis showed a significant reduction in the APP/PS1 mice (205.03 ± 16.88 immunoparticles/µm2) compared to age matched wild type (423.20 ± 20.33 immunoparticles/µm2; Kruskal–Wallis test, pairwise Mann–Whitney U test and Dunn’s method, ***P < 0.001) at 12 months of age. Error bars indicate SEM. Figure S2. Reduced density of synaptic markers in APP/PS1 mice at 12 months. (A‐F) Electron micrographs of the hippocampus showing immunoparticles for the GluN1 subunit of the NMDA receptors, the GIRK2 subunit of GIRK channels and SNP‐25 in the CA1 stratum lacunosum‐moleculare, as detected using the SDS‐FRL technique at 12 months of age. (A,D) Strong immunoreactivity for GluN1 (10 nm gold particles) were observed in postsynaptic membrane specializations in the wild type, while weaker immunoreactivity was detected in the APP/PS1. (G) Quantitative analysis showing a significant reduction of in the APP/PS1 mice (68.74 ± 11.15 immunoparticles/µm2) c [file BPA-30-554-s001.docx]

**Supplementary material**

**Reduction in the neuronal surface of post- and pre-synaptic GABA_B_ receptors in the hippocampus in a mouse model of Alzheimer´s disease**

Martín-Belmonte et al.

**Supplementary Material & Methods**

***Human samples***

Human brain tissue was obtained at autopsy from the Pathologic Anatomy Service of Bellvitge University Hospital (Barcelona, Spain), following national laws and international ethical and technical guidelines on the use of human samples for biomedical research purposes. Sections obtained from of the hippocampal formation of eight individuals were studied (Table 1). Four had clinical and *post mortem* neuropathological diagnoses of AD defined according to Braak and Braak (1991: Table 1). Four were non-demented subjects who did not meet the neuropathologic criteria of AD and are therefore classified as control cases (Table 1). All hippocampal samples were obtained from 3 to 8.5 h after death and were immediately prepared for biochemical studies using western blots. All tissue samples were frozen on dry ice or in liquid nitrogen at time of autopsy and stored at −80ºC until used for analysis. Clinical data for the post-mortem brains used were not available from the brain bank.

# *Antibodies and chemicals*

~~For SDS-FRL, we used rabbit GluA1-4 (pan-AMPA) receptor polyclonal antibody raised against a glutathione~~ *~~S~~*~~-transferase (GST) fusion protein that contained the 58 extracellular amino-acid residues (724–781). The preparation, purification, and full characterization of this antibody, shown to react selectively with all AMPA receptor subunits GluA1–4, were extensively characterized previously (Nusser et al. 1998; Pickard et al. 2000). For western blots, we used a guinea pig pan-AMPA receptor antibody (GP-Af580; aa. 717-754 of mouse AMPA; Frontier Institute Co., Japan). The characteristic of this antibody was described previously (Fukaya et al., 2006).~~

**For SDS-FRL, we used a subunit-specific rabbit GluA1 polyclonal antibodies (AB1505; Millipore Corporation, Burlington, MA, USA) and a subunit-specific rabbit GluA2 polyclonal antibodies (AB1768-I; Millipore Corporation, Burlington, MA, USA).** A monoclonal anti-α-Tubulin (DM1A; ref CP06) was obtained from Millipore (Millipore Corporation, Burlington, MA, USA). A monoclonal antibody against the GluN1 subunit of NMDA receptor was obtained from Millipore (Millipore Corporation, Burlington, MA, USA). The characteristics and specificity of GluN1 was provided previously (Siegel et al., 1994; Iwasato et al., 2000; Masugi-Tokita et al., 2007; Szabadits et al., 2011). A polyclonal anti-GIRK2 (Rb-Af280; aa. 390–421 of mouse GIRK2; Frontier Institute Co., Japan) raised in rabbit was also used. The characteristics and specificity of the anti-GIRK2 antibody have been described elsewhere (Aguado et al. 2008; Fernández-Alacid et al. 2009; Luján et al., 2018). A polyclonal anti-SNAP-25 (ref 111 002; aa 192-206 of Human SNAP-25) raised in rabbit was obtained from Synaptic Systems (Goettingen, Germany; <https://www.sysy.com>) and previously characterised for SDS-FRL (Luján et al., 2018). An antibody against the Rab4 subunit was obtained from BD Transduction Laboratories (ref: 610888), LAMP1 from Enzo Life Sciences (ref: VAM-EN001), EEA1 from BD Transduction Laboratories (ref: 610456); and CHOP from Invitrogen (ref: MA1-250).

The secondary antibodies used were as follows: goat anti-mouse IgG-horseradish peroxidase (1 : 2000; Santa Cruz Biotechnology, Santa Cruz, CA, USA), goat anti-guinea pig IgG-horseradish peroxidase (1 : 2000; Santa Cruz Biotechnology, Santa Cruz, CA, USA), goat anti-rabbit IgG-horseradish peroxidase (1 : 15 000; Pierce, Rockford, USA), anti-mouse IgG conjugated to 10 nm gold particles and anti-rabbit IgG conjugated to 10 nm gold particles (1 : 100; British Biocell International, Cardiff, UK).

***Double-labelling immunofluorescence analysis***

Mice were anaesthetized by intraperitoneal injection of ketamine (0.1 ml/kg b.w.) and xylazine (0.1 ml/kg b.w.) and perfused with 4% paraformaldehyde and 15% (v/v) saturated picric acid made up in 0.1 M phosphate buffer (PB, pH 7.4). After perfusion, brains were dissected and post-fixed in the same fixative at 4°C for 2 hours. Coronal (60 µm) sections were then cut on a Vibratome (Leica V1000) and collected in 0.1 M PB. The sections were then processed further either for double-immunofluorescence procedures. Briefly, free-floating sections were incubated at room temperature with 10% NGS made up in TBS for 1 h before incubation in a mixture of primary antibodies in combination (GABA_B1_ with Rab4 or EEA1 or LAMP1 or CHOP), diluted in TBS containing 1% NGS. Subsequently, after extensive washes in TBS, sections were incubated for 2 hours ~~overnight~~ at 4°C with a mixture of appropriate secondary antibodies. Fluorescence signals were examined using a confocal laser microscope (Leica TLSCM, Austria).

***Quantification and analysis of SDS-FRL data***

The labelled replicas were examined using a transmission electron microscope (JEOL-1010) and images captured at magnifications of 80,000, and 100,000. All antibodies used in this study were visualised by immunoparticles on the exoplasmic face (E-face), consistent with the extracellular location of their epitopes. Non-specific background labelling for pan-AMPA receptors was estimated by counting immunogold particles on the protoplasmic face (P-face) surfaces in wild type mice. Digitized images were then modified for brightness and contrast using Adobe PhotoShop CS5 (Mountain View, CA, USA) to optimize them for quantitative analysis.

*Number and density of AMPA immunoparticles in excitatory synapses.* We determined the number of AMPA immunoparticles composing excitatory synapses present in dendritic spines of CA1 pyramidal cells in the *stratum lacunosum-moleculare* of the CA1 region of the hippocampus, in the two genotypes (wild type and APP/PS1) and the three ages (1, 6 and 12-months). For that purpose, we used the software GPDQ (*Gold Particle Detection and Quantification*) developed recently to perform automated and semi-automated detection of gold particles present in a given compartment of neurons (Luján et al., 2018).

Quantitative analysis of immunogold labelling for AMPA was performed on excitatory postsynaptic specializations indicated by the presence of intramembrane particle clusters (IMP clusters) on the exoplasmic face (E-face) (Harris and Landis, 1986). One of the advantages of the SDS-FRL technique is that the whole synaptic specialisation of excitatory synapses is immediately visible over the surface of neurons. The outline of postsynaptic specialisation (IMP clusters) was manually demarcated by connecting the outermost IMP particles, and the area of synaptic sites was measured using the software GPDQ. Immunogold particles for AMPA were regarded as synaptic labelling if they were within demarcated IMP clusters and those located in the immediate vicinity within 30 nm from the edge of the IMP clusters, given the potential distance between the immunogold particles and antigens (Matsubara et al., 1996). The number of immunogold particles was counted in both complete and incomplete (partially fractured) postsynaptic membrane specialization. Because densities of immunogold labelling for the pan-AMPA antibody obtained from complete and incomplete synapses were not significantly different, they were pooled. The density of the immunoparticles for AMPA in each synaptic site was calculated by dividing the number of the immunoparticles by the area of the demarcated IMP clusters. Measurements were performed in three animals, and results were pooled because the density for immunogold particles was not significantly different in the different animals. Immunoparticle densities were presented as mean ± SEM between animals.

**Supplementary Results**

***Reduction of AMPA receptors in excitatory synapses of 12 months old APP/PS1 mice***

To determine if the effect detected for GABA_B_ receptors in the APP/PS1 mouse model is specific, we analysed as control the distribution of AMPA receptors, known to localise in excitatory synapses in an activity-dependent manner. Using the SDS-FRL method, we determined the numbers and densities of AMPA receptors at excitatory synapses of the *stratum lacunosum-moleculare* (the subfield in which we detected most alteration in the localization of GABA_B_ receptors) in the CA1 region of hippocampal sections with an antibody against highly conserved extracellular amino acid residues of GluA1–4 (pan-AMPA).

Clusters of IMPs on the E-face represent the postsynaptic membrane specialization (PSDs) of glutamatergic synapses (Tarusawa et al., 2009). The analysis performed at 1 month and 6 months of age showed that both in wild type and APP/PS1 mice, immunoparticles for AMPA receptors were distributed over the entire postsynaptic membrane specializations of spines (Suppl Fig. 1A,B,D,E). However, we found similar values in the density of immunoparticles between wild type and APP/PS1 synapses (Suppl Fig. 1G,H). We next investigated the synaptic localisation of AMPA receptors at 12 months of age. In wild type mice, immunoparticles for AMPA receptors in dendritic spines followed a similar synaptic pattern than at earlier ages (Suppl Fig. 1C). In APP/PS1 mice, fewer immunoparticles for AMPA were detected in excitatory synapses of dendritic spines (Suppl Fig. 1F). Although the density of labelling varied between synapses, we found a significant reduction of AMPA receptors in APP/PS1 synapses in the *stratum lacunosum-moleculare* (mean = 205.003 ± 16,87 immunoparticles/µm^2^) compared to age-matched wild type (mean = 407.57 ± 20,32 immunoparticles/µm^2^) (Two-way ANOVA test and Bonferroni *post hoc* test, ***p<0.001) (Suppl Fig. 1I).

***Reduction of signalling molecules in APP/PS1 mice at 12 months of age***

Next, using the same ultrastructural approaches, we analysed as control the distribution of other markers of synapses on spines of pyramidal cells: the GluN1 subunit of the NMDA receptors (which are not really incorporated in an activity dependent manner into synapses), the GIRK2 subunit of G protein-gated inwardly rectifying potassium channels (which are known effector ion channels of GABA_B_ receptors), and the synaptosomal-associated protein SNAP-25 (which is responsible for the calcium-dependent exocytosis of neurotransmitters, thus playing a key role to normal functioning of brain). The analysis was performed in excitatory synapses in the *stratum lacunosum-moleculare* of the hippocampal CA1 region at 12 months of age, the stage in which we found the reduction for AMPA receptors. Immunoparticles for NMDA receptors were distributed over the entire postsynaptic membrane specializations of spines on the exoplasmic face (E-face), consistent with the extracellular location of the epitope (Suppl Fig. 2A,D). We found a significant reduction of NMDA receptors in APP/PS1 synapses in the *stratum lacunosum-moleculare* (mean = 76.08 ± 10.31 immunoparticles/µm^2^) compared to age-matched wild type (mean = 200.75 ± 16.64 immunoparticles/µm^2^) (t test using Holm-Sidak method, ***p<0.001) (Suppl Fig. 2G). Immunoparticles for GIRK2 were detected along the membrane surface (P-face) of dendritic shafts and spines of CA1 pyramidal cells in wild type and APP/PS1 mice (Suppl Fig. 2B,E). Our quantitative analysis demonstrated that immunoreactivity for GIRK2 was significantly reduced in oblique dendrites and spines in APP/PS1 mice (oDen= 92.74 ± 18.12 immunoparticles ⁄μm^2^, *n*=10 dendrites; s= 128.00 ± 12.38 immunoparticles ⁄μm^2^, *n*=9 spines) compared to age-match wild type mice (oDen= 273.10 ± 25.92 immunoparticles ⁄μm^2^, *n*=10 dendrites; s= 461.92 ± 59.88 immunoparticles ⁄μm^2^, *n*=10 spines) (Two-way ANOVA test, ***p<0.001) (Suppl Fig. 2H). Finally, we analysed the possible alteration of SNAP-25, a member of the SNARE complex present exclusively in axons, previously reported to be reduced in human samples with AD (Bereczki et al., 2016) and APP/PS1 model (Yang et al., 2015). Immunoparticles for SNAP-25 were detected along the membrane surface (P-face) of axon terminals in wild type and APP/PS1 mice (Suppl Fig. 2C,F). We observed a significant reduction of SNAP-25 in APP/PS1 axon terminals (mean = 30.73 ± 7.58 immunoparticles/µm^2^) compared to age-matched wild type (mean = 134.02 ± 7.46 immunoparticles/µm^2^) (t test using Holm-Sidak method, ***p<0.001) (Suppl Fig. 2I).

***Co-localization between GABA_B1_ with protein makers***

Given the increase of GABA_B1_ at intracellular sites in APP/PS1 at 12 months of age, we performed experiments to clarify whether these cytoplasmic compartments were endosomes, lysosomes, amphisomes or autophagosomes. For these purpose we used double-labelling immunofluorescence experiments with different maker proteins: 1) EEA1 (early endosomal antigen 1), a marker or early endosomes and amphisomes; 2) Rab4 (small G protein), a marker or early endosomes; 3) LAMP1 (Lysosomal-associated membrane protein 1), a marker of lysosomes; and 4) CHOP (CCAAT-enhancer-binding protein homologous protein), a multifunctional transcription factor involved in Endoplasmic Reticulum (ER) stress (Suppl Fig. 3).

We focused mainly in the somata and proximal dendrites of CA1 pyramidal cells. Immunoreactivity for GABA_B1_ in these compartments was detected as punctate labelling through the cytoplasm surrounding the nuclei of pyramidal cells (Suppl Fig. 3A1-D1, B1-H1). Immunoreactivity for Rab4 (Suppl Fig. 3A2,B2), EEA1 (Suppl Fig. 3C2,D2), LAMP1 (Suppl Fig. 3E2,F2) and CHOP (Suppl Fig. 3G2,H2) were also detected as punctate labelling through the cytoplasm. Although co-localization between GABA_B1_ and the different marker proteins was detected (Suppl Fig. 3A3-H3), no differences in the frequency of co-localization were observed in the APP/PS1 mice compared to age-matched controls.

***Reduction in the expression of GABA_B_ receptors in patients with Alzheimer´s disease***

To validate our data showing the reduction for GABA_B_ receptors detected in the hippocampus in one mouse model, we determined their expression levels together with other markers in human tissue. Thus, using immunoblots we assessed the expression levels of GABA_B1_, ~~pan-AMPA~~, GluA1, GluA2 and GluN1 in tissue lysates of the hippocampus in control and AD cases (Suppl Fig. 4A). In total hippocampal homogenates of all cases, we detected GABA_B1_ as two bands with estimated molecular masses of 130 and 100 kDa, corresponding to the GABA_B1a_ and to the GABA_B1b_ proteins, respectively (Suppl Fig. 4A). Similarly, we detected GluA1and GluA2 as a single predominant band at 100 kDa, and GluN1 as a single predominant band at 120 kDs (Suppl Fig. 4A). Quantification of all these immunoreactive bands revealed that GABA_B1_, GluA1, GluA2 and GluN1 proteins were significantly reduced in the hippocampus of AD subjects compared to controls (Suppl Fig. 4B).

**TABLE 1**. Summary of the main clinical and neuropathological data in the present series.

| *Case Nº* | Gender | Age | PMD (h) | Braak stage | CERAD | Technique |
| --- | --- | --- | --- | --- | --- | --- |
| *1* | Male | 53 | 6.15 | I | 0 | Western blot |
| *2* | Male | 74 | 4.0 | I | 0 | Western blot |
| *3* | Female | 66 | 3.15 | II | A | Western blot |
| *4* | Male | 72 | 8.45 | II | A | Western blot |
| *5* | Male | 74 | 6.0 | V | C | Western blot |
| *6* | Male | 78 | 5.45 | V-VI | C | Western blot |
| *7* | Female | 89 | 5.0 | VI | C | Western blot |
| *8* | Female | 80 | 4.15 | VI | C | Western blot |

Braak Stages (Braak and Braak, 1991): I–II (NFTs in entorhinal cortex and closely related areas); III–IV (NFTs abundant in amygdala and hippocampus and extending slightly into the association cortex); V–VI (NFTs widely distributed throughout the neocortex and ultimately involving primary motor and sensory areas). CERAD Stages (Fillenbaum et al., 2008): A, low density of neuritic plaques; B, intermediate density of neuritic plaques; C, high density of neuritic plaques.

**Figure legends**

**
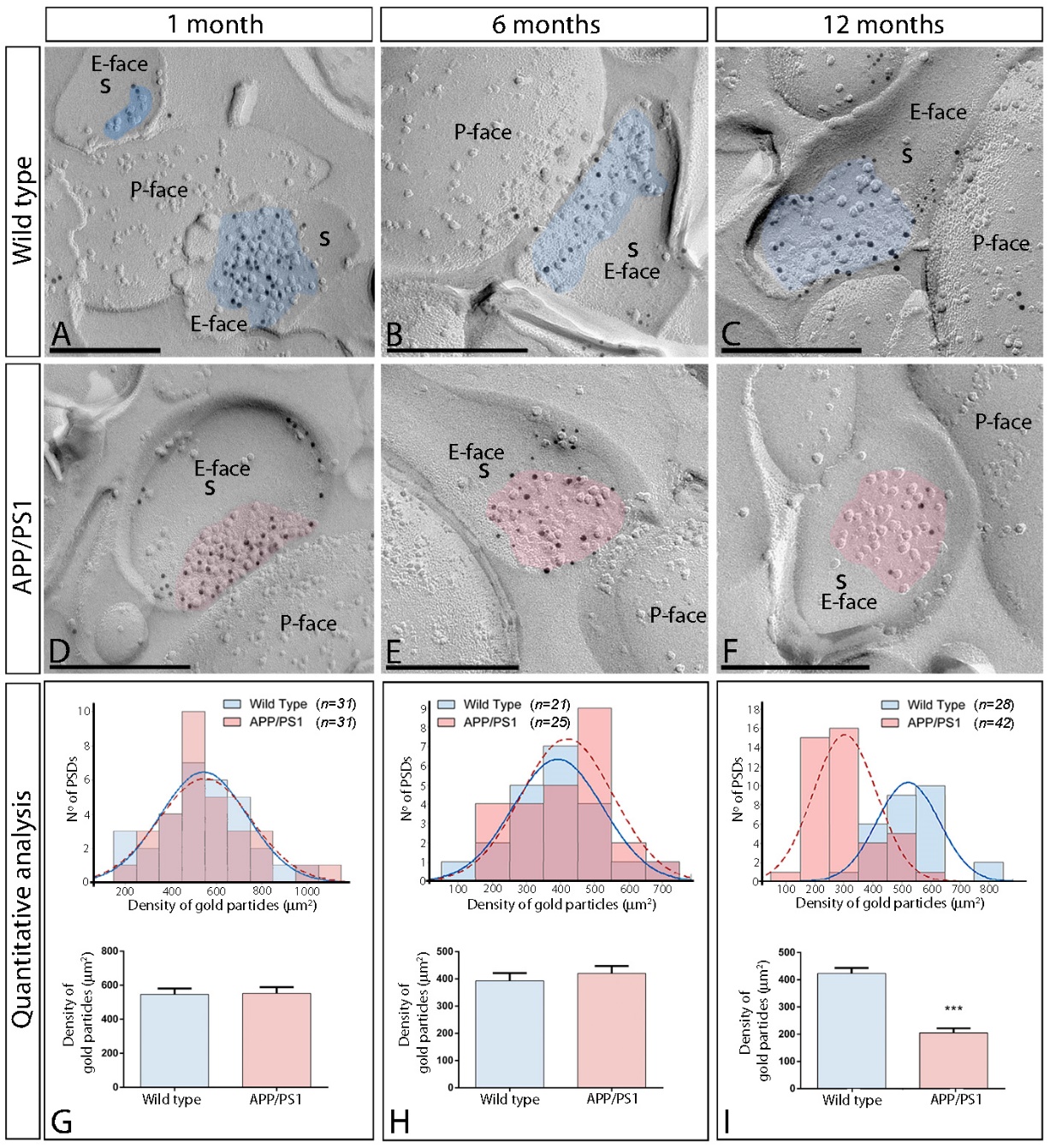
**

**Supplementary Figure 1**. ***Reduced density of synaptic AMPA receptors in dendritic spines of APP/PS1 mice at 12 months***. (A-F) Electron micrographs of the hippocampus showing immunoparticles for pan-AMPA at excitatory synaptic sites of dendritic spines of pyramidal cells in the CA1 *stratum lacunosum-moleculare*, as detected using the SDS-FRL technique at 1, 6 and 12 months of age. Postsynaptic membrane specializations (IMP clusters, pseudo coloured in blue for wild type and in red for APP/PS1 to aid visualization) show strong immunoreactivity for pan-AMPA (10 nm gold particles) in the wild type, while they show weaker immunoreactivity in the APP/PS1. Scale bars: A-F, 200 nm. (G,H,I) Histograms showing the distribution of densities of gold particles that label AMPA receptors in SDS-FRL replicas of individual postsynaptic membrane specializations in wild type and APP/PS1 mice. Quantitative analysis showed no changes in the densities of AMPA immunoparticles in excitatory synapses in dendritic spines at 1 and 6 months of age (panels G and H). However, the analysis showed a significant reduction in the APP/PS1 mice (205.03 ± 16.88 immunoparticles/µm^2^) compared to age matched wild type (423.20 ± 20.33 immunoparticles/µm^2^; Kruskal–Wallis test, pairwise Mann–Whitney U test and Dunn’s method, ***p<0.0001) at 12 months of age. Error bars indicate SEM.


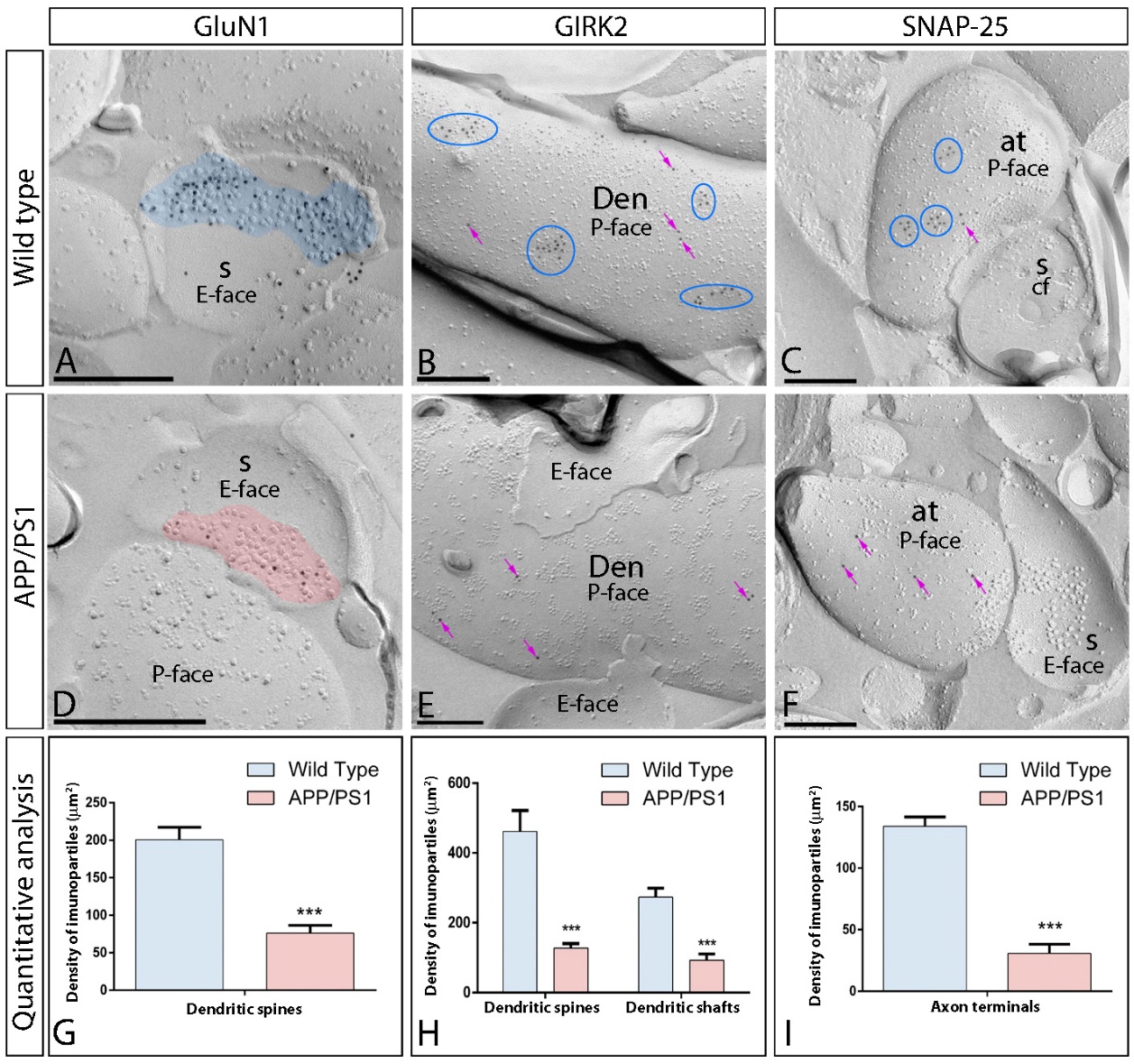


**Supplementary Figure 2**. ***Reduced density of synaptic markers in APP/PS1 mice at 12 months***. (A-F) Electron micrographs of the hippocampus showing immunoparticles for the GluN1 subunit of the NMDA receptors, the GIRK2 subunit of GIRK channels and SNP-25 in the CA1 *stratum lacunosum-moleculare*, as detected using the SDS-FRL technique at 12 months of age. (A,D) Strong immunoreactivity for GluN1 (10 nm gold particles) were observed in postsynaptic membrane specializations in the wild type, while weaker immunoreactivity was detected in the APP/PS1. (G) Quantitative analysis showing a significant reduction of in the APP/PS1 mice (**68.74 ± 11.15 immunoparticles/µm^2^) compared to age matched wild type (198.56 ± 15.24** immunoparticles/µm^2^; Kruskal–Wallis test, pairwise Mann–Whitney U test and Dunn’s method, ***p<0.0001). (B,E) Immunoparticles for GIRK2 were observed along the surface membrane of dendrites and spines. (H) Quantitative analysis showing that immunoreactivity for GIRK2 was significantly reduced in oblique dendrites (oDen) and spines in APP/PS1 mice (oDen= 92.74 ± 18.12 immunoparticles ⁄μm^2^, *n*=10 dendrites; s= 128.00 ± 12.38 immunoparticles ⁄μm^2^, *n*=9 spines) compared to age-match wild type mice oDen= 273.10 ± 25.92 immunoparticles ⁄μm^2^, *n*=10 dendrites; s= 461.92 ± 59.88 immunoparticles ⁄μm^2^, *n*=10 spines) (Two-way ANOVA test, *p < 0.05; ***p<0.001). (C,F) Immunoreactivity for SNAP-25 were detected along the extrasynaptic plasma membrane of axon terminals (at) facing dendritic spines (s). (I) Our quantitative analysis showed a significant reduction in the density of SNAP-25 in the APP/PS1 mice (30.73 ± 7.58 immunoparticles/µm^2^) compared to age matched wild type (mean = 134.02 ± 7.46 immunoparticles/µm^2^; t test using Holm-Sidak method, ***p<0.001).. Scale bars: A-F, 200 nm. Error bars indicate SEM.


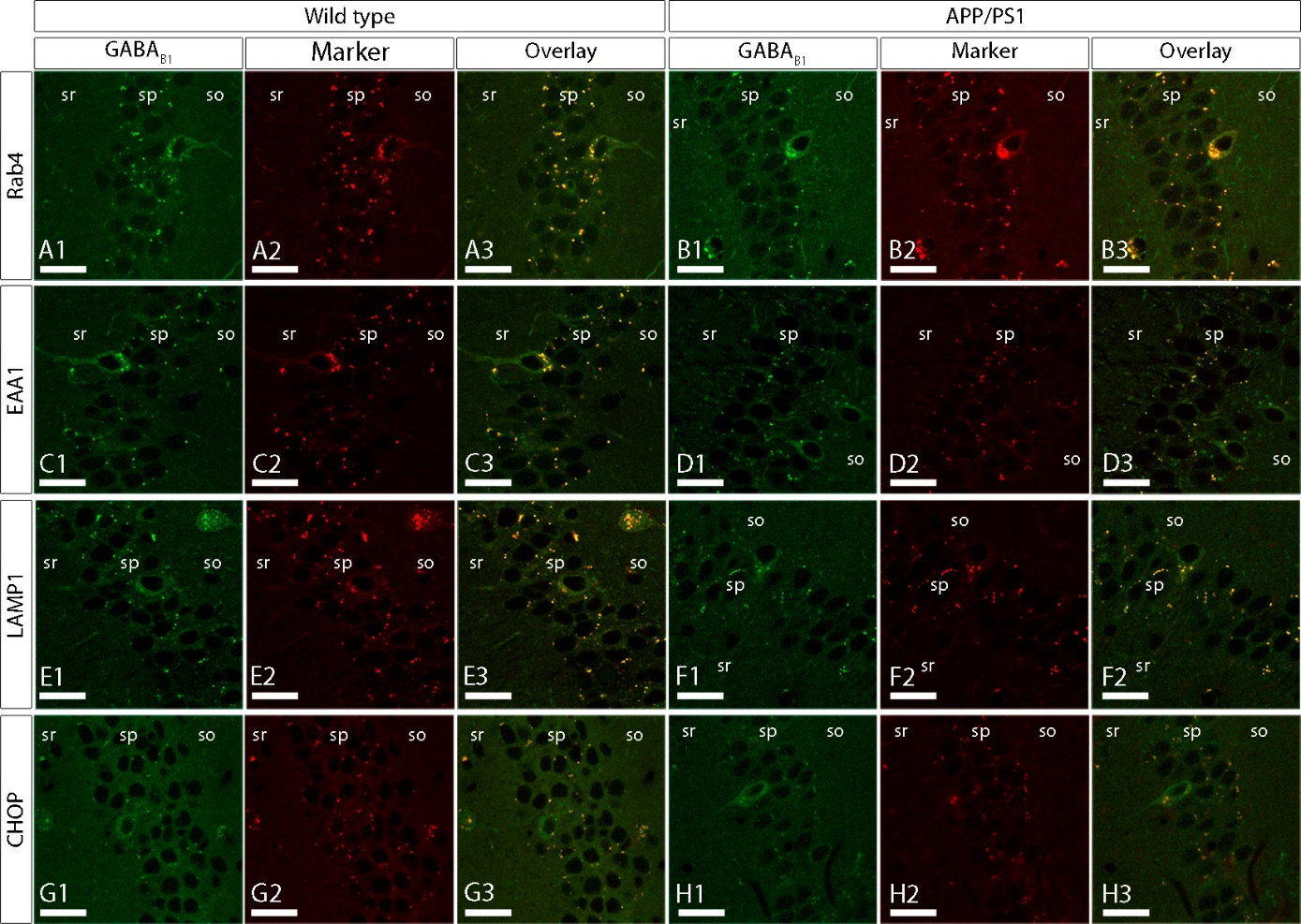


**Supplementary Figure 3**. ***Co-localization of GABA_B_ receptors with marker proteins*** ***in APP/PS1 mice at 12 months***. (A-H) Immunofluorescence for GABA_B1_ (green) and Rab4 (red), EEA1 (red), LAMP1 (red) or CHOP (red) in wild type and APP/PS1 mice at 12 months of age. The overlay between GABA_B1_ and each marker protein can be seen in yellow (A3-G3, B3-H3). Co-localization between GABA_B1_ and the different marker proteins was detected mainly in the somata of pyramidal cells in the *stratum pyramidale*, but no differences in the frequency of co-localization was observed in the APP/PS1 mice compared to age-matched controls. so, *stratum oriens*; sr, *stratum radiatum*. Scale bars: A-H, 20 µm.


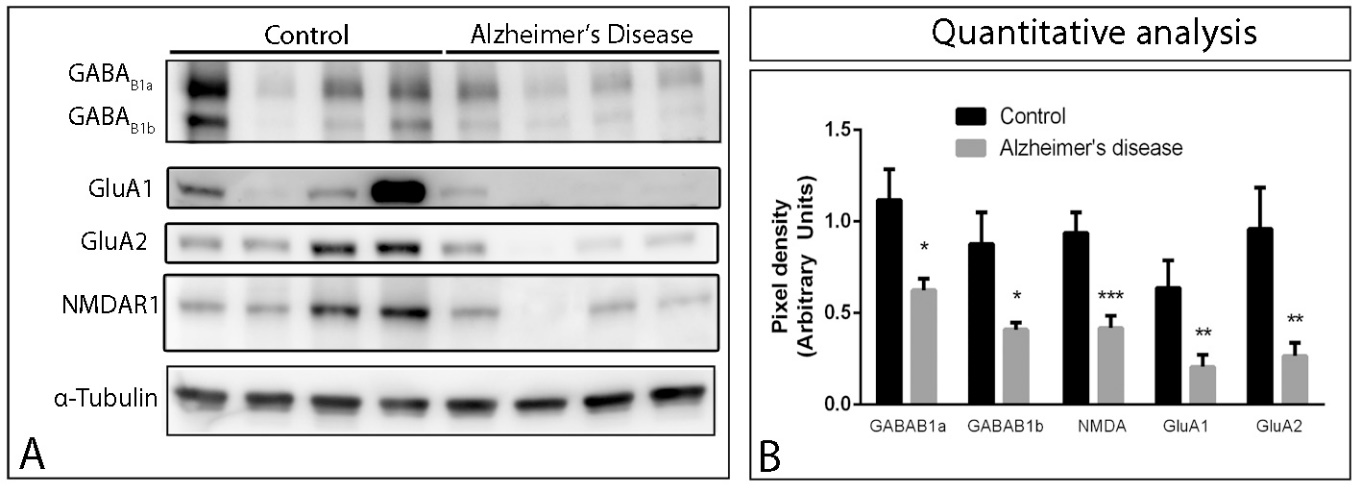


**Supplementary Figure 4. *Representative western blots of different molecules in the hippocampus from control and Alzheimer´s patients***. (A) Crude membrane preparations were subjected to 5% SDS-PAGE, transferred on to polyvinylidene difluoride membranes. They were reacted with an anti-pan GABA_B1_ antibody, which recognised both GABA_B1a_ and GABA_B1b_ subunits with estimated molecular masses of 130 and 100 kDa, respectively. The antibodies to GluA1, GluA2 detected a single predominant band at 100 kDa and NMDA detected a single predominant band at 120 kDs. (B) The developed immunoblots were scanned and densitometric measurements were averaged together to compare the protein densities between controls and AD in the hippocampus. Quantification of GABA_B1_, GluA1, GluA2 and GluN1 normalised to α-tubulin and expressed as pixel density showed a significant reduction in the amount of proteins when compared AD with controls. An antibody anti α-Tubulin served as an internal control to ensure comparable protein loading showed no significant difference among AD or control groups. Data are means ± SEM of represented cases. *p<0.05; **p<0.01; ***p<0.001.


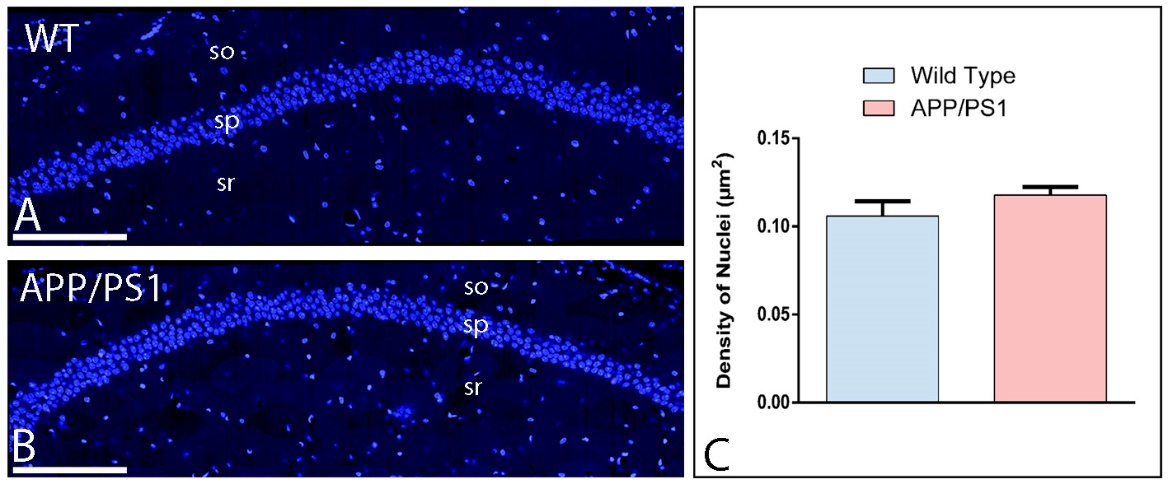


**Supplementary Figure 5**. ***The density of CA1 pyramidal cells is not altered*** ***in APP/PS1 mice at 12 months***. (A) Hippocampal sections from WT and APP/PS1 were stained with DAPI. (B) Quantification of the number of nuclei in the pyramidal cell layer of the CA1 region. We confirmed that the number of neurons is similar between WT and APP/PS1. Scale bars: A-B, 100 µm.

**Bibliography**

Aguado C, Colon J, Ciruela F, Schlaudraff F, Cabanero MJ, Perry C, Watanabe M, Liss B, Wickman K, Lujan R (2008) Cell typespecific subunit composition of G protein-gated potassium channels in the cerebellum. J Neurochem 105:497–511.

Bereczki E, Francis PT, Howlett D, Pereira JB, Höglund K, Bogstedt A, Cedazo-Minguez A, Baek JH, Hortobágyi T, Attems J, Ballard C, Aarsland D. (2016) Synaptic proteins predict cognitive decline in Alzheimer's disease and Lewy body dementia. Alzheimers Dement. 2016 Nov;12(11):1149-1158.

Braak H, Braak E (1991) Neuropathological stageing of Alzheimerrelated changes. Acta Neuropathol 82:239–259.

Fernandez-Alacid L, Aguado C, Ciruela F, Martın R, Colon J, Cabanero MJ, Gassmann M, Watanabe M, Shigemoto R,Wickman K, Bettler B, Sanchez-Prieto J, Lujan R (2009) Subcellular compartment-specific molecular diversity of pre- and post-synaptic GABA-activated GIRK channels in Purkinje cells. J Neurochem 110:1363–1376.

Fillenbaum GG, Belle G Van, Morris JC, Richard C, Mirra SS, Davis PC, Tariot PN, Silverman M, Clark CM, Welsh-Bohmer KA, Heyman A (2008) CERAD (Consortium to Establish a Registry for Alzheimer’s Disease): The first 20 years. *Alzheimers Dement* **4**, 96-109.

Fukaya M, Tsujita M, Yamazaki M, Kushiya E, Abe M, Akashi K, Natsume R, Kano M, Kamiya H, Watanabe M, Sakimura K. 2006. Abundant distribution of TARP gamma-8 in synaptic and extrasynaptic surface of hippocampal neurons and its major role in AMPA receptor expression on spines and dendrites. Eur J Neurosci. 24(8):2177-90.

Harris KM, LandisDM (1986) Membrane structure at synaptic junctions in area CA1 of the rat hippocampus. Neuroscience 19:857– 872.

Iwasato T, Datwani A, Wolf, AM, Nishiyama H, Taguchi Y, Tonegawa S, Knopfel T, Erzurumlu RS, Itohara S. 2000. Cortex-restricted disruption of NMDAR1 impairs neuronal patterns in the barrel cortex. Nature 406:726–731.

Luján R, Aguado C, Ciruela F, Cózar J, Kleindienst D, de la Ossa L, Bettler B, Wickman K, Watanabe M, Shigemoto R, Fukazawa Y. 2018. Differential association of GABA_B_ receptors with their effector ion channels in Purkinje cells. Brain Struct Funct. 223(3):1565-1587.

Masugi-Tokita M, Tarusawa E, Watanabe M, Molnar E, Fujimoto K, Shigemoto R (2007) Number and density of AMPA receptors in individual synapses in the rat cerebellum as revealed by SDS-digested freeze-fracture replica labeling. J Neurosci 27:2135–2144.

Matsubara A, Laake JH, Davanger S, Usami S, Ottersen OP (1996) Organization of AMPA receptor subunits at a glutamate synapse: a quantitative immunogold analysis of hair cell synapses in the rat organ of Corti. J Neurosci 16:4457–4467.

Nusser Z, Lujan R, Laube G, Roberts JDB, Molnar E, Somogyi P (1998) Cell type and pathway dependence of synaptic AMPA receptor number and variability in the hippocampus. Neuron 21:545–559.

Pickard L, Noël J, Henley JM, Collingridge GL, Molnar E (2000) Developmental changes in synaptic AMPA and NMDA receptor distribution and AMPA receptor subunit composition in living hippocampal neurons. J Neurosci 20:7922–7931.

Siegel SJ, Brose N, Janssen WG, Gasic GP, Jahn R, Heinemann SF, Morrison JH (1994) Regional, cellular, and ultrastructural distribution of *N-*methyl-D-aspartate receptor subunit 1 in monkey hippocampus. Proc Natl Acad Sci U S A 91:564 –568.

Szabadits E, Cserep C, Szonyi A, Fukazawa Y, Shigemoto R, Watanabe M, Itohara S, Freund TF, Nyiri G. 2011. NMDA receptors in hippocampal GABAergic synapses and their role in nitric oxide signaling. J Neurosci 31: 5893–5904.

Tarusawa E, Matsui K, Budisantoso T, Moln´ar E,Watanabe M, Matsui M, Fukazawa Y, Shigemoto R. (2009). Input-specific intrasynaptic arrangements of ionotropic glutamate receptors and their impact on postsynaptic responses. J Neurosci 29, 12896–12908.

Yang Y, Kim J, Kim HY, Ryoo N, Lee S, Kim Y, Rhim H, Shin YK. (2015) Amyloid-β Oligomers May Impair SNARE-Mediated Exocytosis by Direct Binding to Syntaxin 1a. Cell Rep. 12(8):1244-51.
